# Field evidence of bird poisonings by imidacloprid-treated seeds: a review of incidents reported by the French SAGIR network from 1995 to 2014

**DOI:** 10.1007/s11356-016-8272-y

**Published:** 2016-12-27

**Authors:** Florian Millot, Anouk Decors, Olivier Mastain, Thomas Quintaine, Philippe Berny, Danièle Vey, Romain Lasseur, Elisabeth Bro

**Affiliations:** 1 Research Department, National Game and Wildlife Agency, Office National de la Chasse et de la Faune Sauvage (ONCFS), 5 rue de Saint-Thibaut, 78610 Saint Benoist, Auffargis France; 2 Biodiversity and Water Department, French Ministry of Environment, Energy and Sea (MEEM), 92400 La Défense, France; 3 College of Veterinary Medicine, Toxicology, Ecole Nationale Vétérinaire de Lyon, 1, av Bourgelat, 69280 Marcy l’étoile, France

**Keywords:** Imidacloprid, Dressed seeds, Birds, Mortality, In situ, Wildlife

## Abstract

The large-scale use of neonicotinoid insecticides has raised growing concerns about their potential adverse effects on farmland birds, and more generally on biodiversity. Imidacloprid, the first neonicotinoid commercialized, has been identified as posing a risk for seed-eating birds when it is used as seed treatment of some crops since the consumption of a few dressed seeds could cause mortality. But evidence of direct effects in the field is lacking. Here, we reviewed the 103 wildlife mortality incidents reported by the French SAGIR Network from 1995 to 2014, for which toxicological analyses detected imidacloprid residues. One hundred and one incidents totalling at least 734 dead animals were consistent with an agricultural use as seed treatment. Grey partridges (*Perdix perdix*) and “pigeons” (*Columba palumbus*, *Columba* *livia* and *Columba oenas*) were the main species found. More than 70% of incidents occurred during autumn cereal sowings. Furthermore, since there is no biomarker for diagnosing neonicotinoid poisonings, we developed a diagnostic approach to estimate the degree of certainty that these mortalities were due to imidacloprid poisoning. By this way, the probability that mortality was due to poisoning by imidacloprid-treated seeds was ranked as at least “likely” in 70% of incidents. As a result, this work provides clear evidence to risk managers that lethal effects due to the consumption by birds of imidacloprid-treated seeds regularly occur in the field. This in turn raises the question of the effectiveness of the two main factors (seed burying and imidacloprid-treated seeds avoidance) that are supposed to make the risk to birds negligible. Risk factors and the relevance of mitigation measures are discussed.

**Electronic supplementary material:**

The online version of this article (doi:10.1007/s11356-016-8272-y) contains supplementary material, which is available to authorized users.

## Introduction

The large-scale use of neonicotinoids has raised growing concerns about their potential adverse effects on nontarget invertebrates especially on pollinators (see Pisa et al. 2014 for a review). By the way, in December 2013, the European Union banned the use of three neonicotinoid insecticides (clothianidin, imidacloprid and thiametoxam) for seed coating, soil treatment and foliar treatment on crops attractive to pollinators. Nevertheless, their use after crop flowering, or on crops harvested before flowering, and for seed treatment of winter cereals continues to be approved (Regulation 485/2013).

Neonicotinoids are insecticides that act as nicotinic acetylcholine receptor (nAChR) agonists. They have a greater affinity for insect nAChRs than to those of vertebrates (Tomizawa and Casida 2005). As a result, they are generally thought to be less harmful to mammals and birds. However, concerns for vertebrates, especially birds, have also been raised. In a recent correlative study, Hallmann et al. (2014) suggested that neonicotinoids may have a more important impact on bird populations than previously suspected. They showed that birds have declined faster in areas with higher neonicotinoid concentrations in surface water. Because of the low toxicity of neonicotinoids to vertebrates and the diet of birds studied (mainly insectivorous), these authors argued that it is more likely that the observed declines are the result of knock-on effects of the widespread depletion of the insect populations caused by neonicotinoids. Nonetheless, as their results are derived from correlation, adverse consequences on bird populations due to direct lethal or sublethal effects cannot totally be excluded. All the more so that, at concentrations relevant to field exposure scenarios, neonicotinoids have the potential to cause direct adverse effects (e.g. Gibbons et al. 2015; Goulson 2013; Lopez-Antia et al. 2013, 2015; Mineau and Palmer 2013). A few papers reported wild bird mortalities due to neonicotinoid poisoning (Berny et al. 1999; Bro et al. 2004, Bro et al. 2010) with for some cases the observation of sublethal effects (Bro et al. 2010). Yet, field evidence of neonicotinoid direct toxic effects on birds remains scarce. For this purpose, we reviewed wildlife incidents reported by the French SAGIR Network (national network for the surveillance of the health status of wildlife) from the 1 January 1995 to the 31 December 2014 and for which residue of neonicotinoids were detected.

Here, we focused on imidacloprid. Imidacloprid has been the first commercialized neonicotinoid and it was, in 2009, the largest selling insecticide in the world accounting for 41.5% of the whole neonicotinoid market (Jeschke et al. 2011). Imidacloprid oral median lethal dose (LD50) for birds varies from 13,9 mg/kg for the grey partridge (*Perdix perdix*) to 283 mg/kg for the mallard (*Anas platyrhynchos*), which classifies it as highly toxic to some birds according to the US Environmental Protection Agency (EPA) classification (see Gibbons et al. 2015 for review). Furthermore, sublethal effects on birds as reproductive impairment or abnormal behaviour occur at much lower concentrations than lethal effects (Gibbons et al. 2015). In agriculture, imidacloprid is mainly used as seed treatment in a broad range of crops (Goulson 2013; Jeschke et al. 2011). As a seed treatment, it has been identified as a risk for granivorous birds (Gibbons et al. 2015; Goulson 2013, Mineau and Palmer 2013). Indeed, the ingestion of even a few treated seeds could cause mortality or sublethal effects to sensitive bird species (Gibbons et al. 2015; Mineau and Palmer 2013). For example, for a grey partridge weighing approximately 390 g, the ingestion of just six beet seeds are necessary to reach the LD50 (Goulson 2013).

Two main factors are supposed to reduce the bird exposure to treated seeds which explain, in part, that the risk for granivorous birds posed by the use of imidacloprid as seed treatment is considered as low by risk assessors (e.g. ANSES 2011; EFSA 2008). The first factor is that seeds are supposed to be buried below the soil surface during sowing, making them less or not accessible for granivorous birds. The second one is the avoidance by birds of imidacloprid-treated seeds (Avery et al. 1993, Avery et al. 1994; Lopez-Antia et al. 2014). Nonetheless, as wild bird poisoning due to the ingestion of imidacloprid-treated seeds have already been detected in the field (Berny et al. 1999; Bro et al. 2004, Bro et al. 2010), these mitigating measures seem not to allow a total protection at least under some circumstances and for some birds.

In this context, this work has three main objectives. The first one is to report that mortality events of granivorous birds associated with a real exposure to imidacloprid-treated seeds are regularly recorded in the field despite the two main factors assumed to significantly reduce the probability of exposure to these treated seeds. The second objective is to assess whether imidacloprid poisoning may have been the cause of these casualties. In contrast to cholinesterase inhibitors, there is no biomarker to investigate neonicotinoid poisonings (Mineau and Palmer 2013). So, we used some eco-epidemiological criteria and a weight of evidence approach to (i) establish the link between the clinical effect (i.e. the mortality) and the exposition to imidacloprid-treated seeds and (ii) estimate the intensity of the link. The third objective is to discuss whether the occurrence of these incidents may be the result of particular ecological or agricultural circumstances and whether they correspond to either exceptional or recurring facts.

As a result, this work provides “real-life data” to risk managers about the direct effects of agricultural use of imidacloprid as seed treatments on granivorous birds to sustain decision-making procedures.

## Material and methods

### Functioning of the SAGIR network

In France, the surveillance of pesticide unexpected acute effects on free-ranging wild birds and mammals is performed through the national network of epidemiological surveillance “SAGIR”. SAGIR is a generalist incident-based surveillance network for epidemiological vigilance towards wildlife disease dealing with early detection and early warning. In other words, SAGIR aims at detecting, as early as possible, abnormal mortality or morbidity signals and investigating the aetiologies of the ongoing morbid process. The vigilance relies on a diagnostic process, based on a transdisciplinary approach (epidemiology, ecology, toxicology and pathology) guided by field clues, post-mortem examinations, and scientific expertise acquired over time.

In practical terms, two professional technicians coordinate together in each French department (French administrative area) various observers - not only including professionals but also hunters, naturalists and farmers, -which can report mortality events to the SAGIR network. The data collection process can be considered as an opportunistic sampling (Dohoo et al. 2003) as only the most conspicuous carcasses are likely to be detected. A wide array of mammal and bird species are routinely collected: brown hare (*Lepus europaeus*), rabbit (*Oryctolagus cuniculus*), roe deer (*Capreolus capreolus*), wild boar (*Sus scrofa*), red fox (*Vulpes vulpes*), grey partridge, red-legged partridge (*Alectoris rufa*), ring-necked pheasant (*Phasianus colchicus*), pigeon sp. (of which *Columba palumbus*), raptors (of which kites *Milvus milvus* and *Milvus nigra*, buzzard *Buteo buteo*, tawny owl *Strix aluco*) and a wide array of passerines (e.g. Alaudidae, Corvidae, Embrizidae, Fringillidae, Turdidae, Sturnidae), etc. (Decors et al. 2011).

For each mortality event collected within the SAGIR network, an individually numbered form is filled in to notify the epidemiological, agricultural and ecological circumstances associated with the discovery, as well as the clinical signs observed on the carcass(es). The form ensures the traceability of the results during the entire SAGIR process. When possible, moribund animals with evident clinical signs are filmed to be characterized by a veterinarian. Then fresh, chilled or frozen carcasses and their associated forms are submitted to the local administrative laboratory of veterinary analyses for post-mortem examination. The data specified in the form guide the veterinarians and the toxicologist when implementing necropsy and residue analysis.

### Necropsy and residue analysis

A gross pathologic examination was performed on each submitted animal. According to field and epidemiological clues as well as gross pathologic and clinical picture, additional tests (parasitology, bacteriology, virology, histology) helpful to investigate the aetiology of death were prioritized. These tests included residue analyses.

Residue analyses were set up whenever epidemiology or agricultural contexts were consistent with poisoning. So, there were no systematic procedure (i.e. a screen of an array of predefined substances) implemented when a case of poisoning was suspected. They were performed by the Toxicological Laboratory of the Veterinary School VetAgro sup (Lyon, France). Active substances were targeted on the basis of the agricultural context (crop, phenological stage, kind of treatment, etc.), their potential acute toxicity, the observed effect, and the scientific expertise of the toxicologist. To date, 67 different active substances of pesticides have been searched for at least in one incident recorded by the SAGIR. Yet, four main types of pesticides gather 73% of total residue analyses: cholinesterase inhibitors (34%), organochlorins (19%), neonicotinoids (15%), and anticoagulant (13%) (see Berny and Gaillet 2008 for details of substances usually searched for in the SAGIR and the associated methods).

Regarding imidacloprid, while residue analyses may have been performed all year long, they were mainly focused during the period of agricultural utilization of imidacloprid—especially during crop sowing periods—in case of discovery of dead (with or without predation signs), or moribund animals showing signs of neurological disturbance, especially when these animals were found close to fields recently sown.

A specific detection with a high performance thin layer chromatography, using UV detection was conducted to identify and quantify the compounds, with a limit of detection of 0.1 μg/g of fresh tissue for both crop/gizzard and liver. Confirmation was made with a high performance liquid chromatography, using UV detection (Berny et al. 1999). Only the parent compound was targeted but not its active metabolites (in particular, 6-chloronicotinic acid) as our concern was acute poisoning. The fraction of metabolites was also considered as being in small quantity, based on the laboratory experience with former imidacloprid exposure cases (Berny et al. 1999). As the lethal action of imidacloprid could be faster than its distribution in tissues (Thyssen and Machemer 1999), crop/gizzard contents were analysed first, but hepatic levels were also determined as far as possible.

When mortality events involved several animals, a sample of individuals was collected for necropsy and residue analyses as long as the circumstances of the event were unchanged. Sometimes, for cost reasons, we performed a necropsy on each individual collected but individuals were pooled for residues analyses. This was the case for approximately 46% of incidents involving several animals.

### Data analysis

In the SAGIR, an incident was defined as a mortality event involving one or more individuals discovered during a short period (e.g. the same sowing period) in a limited area (e.g. the same municipality) and for which the ecological, epidemiological and agricultural circumstances as well as the clinical signs are the same.

For the purpose of this work, all incidents associated with a real exposure to imidacloprid (i.e. residues of imidacloprid were detected in crop/gizzard content and/or in liver of at least one carcass) that occurred between 1 January 1995 and 31 December 2014 were extracted from the SAGIR national database. Such cases are hereafter called “incidents” or “imidacloprid-confirmed incidents”.

For each incident, we reviewed the number of animals involved, the spatial and temporal occurrence, the field circumstances, and the results of post-mortem examination and residue analyses. We defined an incident with a minimum of two animals discovered at the same sowing period in the same municipality as a clustered incident.

We described more precisely post-mortem examinations and residue analysis results for the grey partridge and “pigeons” (gathering *C*. *palumbus, Columba livia* and *Columba oenas* ) given that they represented 88% of the sample and that they have biological differences that are important with regard to ecological risk assessment (see “Discussion” section). We used the Fisher exact test to investigate for differences in the types of lesions (predation marks, congestion, and haemorrhage) or other clinical signs (nervous disorders) observed in grey partridges and pigeons. The differences of imidacloprid concentrations dosed in crop/gizzard and liver between grey partridges and pigeons were tested with the Mann-Whitney *U* test. For this analysis, we took into account only carcasses with positive results (i.e. concentration above the detection limit).

The detection of incidents was not based on a pre-defined sampling process, thus interpreting the spatial and temporal variability of incidents should be cautious. However, given that we pooled a 20-year data, we investigated the spatiotemporal coincidence between the occurrence of these incidents and the use of imidacloprid as seed treatment in order to assess the strength of this association (Fox 1991; Hewitt et al. 2003). In addition, we studied two points in particular.

Firstly, in France, the use of imidacloprid as seed treatment of sunflower and maize was banned in 1999 and in 2004, respectively. So, we examined the influence of these bans through the seasonal distribution of incidents. But, as no incident has been associated with the ingestion of imidacloprid-treated sunflower seeds (no sunflower seed was reported in crop/gizzard contents of carcasses and no incident was reported on recently sown sunflower fields, see “Results” section) we looked only at the influence of the ban on maize seeds. Here, we make the assumption that the proportion of confirmed imidacloprid detections (i.e. imidacloprid-confirmed incidents) among the total number of incident for which imidacloprid residue analysis has been performed (hereafter called “imidacloprid-analysed incidents”) have decreased after 2004 during the maize sowing season (April to June). In order to assess that a decrease in the proportion of imidacloprid detection could not be due to other factors, as overall decline of the use of imidacloprid as seed treatments, we also tested this assumption for the other seasons. For this purpose, we used the Fischer exact test.

Secondly, we wanted to assess whether the occurrence of imidacloprid incidents was proportional to the use of imidacloprid-treated seeds. To investigate thoroughly this assumption, spatiotemporal information about the use of imidacloprid-treated seeds would be helpful but this kind of data is not readily accessible in France. So, we used the acreage of crops for which imidacloprid is authorized as seed treatment instead of imidacloprid-use quantitative data. For this purpose, we examined the evolution of the proportion of imidacloprid-confirmed incident among the total suspected poisoning incidents according to the acreage of winter cereals (winter wheat and barley) in French administrative departments. We took as denominator the total suspected poisoning incidents reported by SAGIR (rather than only the imidacloprid-analysed incidents) in order to take into account also the incident for which a suspicion of imidacloprid has been ruled out during the SAGIR process before the step of residue analysis. Indeed, these incidents can be considered as “negative” results regarding imidacloprid detection. We focused only on autumn incidents (from September to December) because, in spring, the diversity of imidacloprid-treated crops was wider, with overlapping sowing periods and fewer incidents were reported. Moreover, we focused on pigeons and partridges suspected poisoning incidents in order to reduce potential sources of spatial bias in the detection and reporting of incidents of different species (see “Discussion” section). For each French department, we calculated the average acreage of winter cereals from 1995 to 2014 (data from the statistical and forecasting office of the French ministry of agriculture, http://agreste.agriculture.gouv.fr). Then, we classified the departments in five classes for the winter cereal acreage (≤100 km^2^, ]100–500 km^2^], ]500–1000 km^2^], ]1000–1500 km^2^], >1500 km^2^). Next, we cumulated all the incidents of all departments belonging to a given class and calculated the proportion of incidents with confirmed imidacloprid detection. Finally, for each proportion we estimated the 95% confidence interval by the Clopper-Pearson method (Clopper and Pearson 1934).

Statistical analyses were performed using the computing environment R (R Core Team 2015). The additional package “PropCIs” (Scherer 2014) were used for estimating the 95% confidence interval by the Clopper-Pearson method.

### Diagnosis of poisoning

**Fig. 1:**
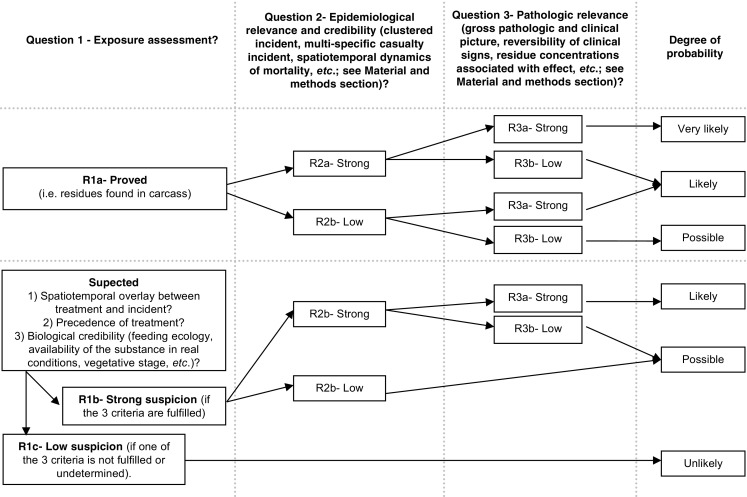
Decision tree to estimate the probability that an incident is due to pesticide poisoning (modified from Aubertot et al. (2005); Forbes and Calow 2002, Forbes and Calow 2004)

A diagnosis of poisoning relies usually on the acute toxicity of the suspected substance, the certainty of exposure, the amount of chemical ingested and the relevance of the clinical picture. This information is often partial regarding wildlife casualties or references are absent. For example, acute toxicity laboratory tests (e.g. LD50) are rarely available for wildlife species. Approaches, combining the use on eco-epidemiological criteria (Fox 1991; Forbes et Callow 2002; Hewit et al. 2003) and a weight-of-evidence approach to integrate the totality of these criteria (e.g. Forbes et Callow 2002, 2004) have been developed to assess whether causal relationships exist between the presence in the environment of contaminants and observed effects, especially when data are partial or not sufficient to be strictly demonstrative. Based on these works, in the SAGIR network, we developed a weight-of-evidence approach to establish the diagnosis of poisoning. This approach is summarized in a general decision tree (Fig. 1) whose aims are both to make the criteria used to establish the diagnosis of poisoning the most objective as possible and to give a qualitative judgement on the probability of a causal link by classifying it into four categories: “unlikely”, “possible”, “likely”, and “very likely”. This diagnosis takes place at the end of the SAGIR process when all post-mortem examinations and analyses have been performed. It can evolve as scientific expertise develops or new knowledge comes to light. Moreover, the answers of each question can be adapted for each group of substances (e.g. cholinesterase inhibitors, neonicotinoids, anticoagulant, etc.).

The first eco-epidemiological criterion used to establish the diagnosis of poisoning is the exposure to the substance. In the field, there are two kinds of situations depending on whether the exposure can be investigated by residue analyses or not (Fig. 1). Indeed, suitable toxicological analyses are not always feasible because of toxicokinetic (rapid degradation of the substance in the carcass) or biologic (scavenged carcass) reasons. In this case, the exposure is assessed through the spatiotemporal coincidence of the use of the substance and the occurrence of the incident, as well as the biological credibility of the exposure (feeding ecology, etc.). In this work, we focused only on incidents for which imidacloprid residue have been detected in order to reduce the potential source of uncertainty.

The second criterion is the epidemiological relevance of the poisoning. This was achieved by studying the field circumstances associated with the discovery of the incident (timing of mortality, discovery area, etc.). For example, the discovery of several individuals all died at once, in good body condition, and found in crops less than a few meters apart suggest a hyperacute pathological process. In addition, this temporal distribution is consistent with a traumatic or toxic aetiology. The spatial aggregation may provide information on the source of contamination, especially if it might be a food-borne disease. So, we classified as “strong” epidemiological credibility (answer R2a, Fig. 1) the clustered incidents with an epizootic curve consistent with poisoning (i.e. animals dead the same day and found close each other), provided that they were discovered during the sowing period in a crop area where imidacloprid-treated seeds were used. Single animal incidents lack sufficient epidemiological evidence that clearly points to pesticide poisoning.

The third criterion is the pathologic relevance of the poisoning. This was based on the review of clinical signs, post-mortem examinations, residue analysis and exclusion of the other most evident causes of hyperacute process. In the case of a hyperacute pathologic process (as an acute poisoning) macroscopic lesions are often scarce and when present, toxicant-induced lesions are often nonspecific. In addition, clinical signs of pesticide poisoning are rarely distinctive of one substance. Thus**,** the absence of macroscopic lesion or the presence of nonspecific lesions (e.g. generalized congestion) in birds in good body condition suggest an acute pathologic process. Regarding imidacloprid, nervous disorders as sudden fall in flight, and ataxia (see “Results” section) are not specific but compatible with the nicotinic action of imidacloprid. Indeed, Cox (2001) reported in-coordination and inability to fly in house sparrow exposed to a dose of 6 mg/kg of imidacloprid. Avery et al. (1993) observed ataxia in red-winged blackbirds (*Agelaius phoeniceus*) and brown-headed cowbirds (*Molothrus ater*) that fed on imidacloprid-treated seeds; however, this effect was quickly reversible. So, the observation of nervous transitory effects is also consistent with an imidacloprid poisoning. Regarding the residue analysis results, the liver is the most reliable organ to confirm a suspected imidacloprid poisoning case (Berny et al. 1999). Indeed, the concentration of imidacloprid in the crop/gizzard contents may be unreliable and lead to false conclusions. For example, if the bird regurgitated part of its meal as a consequence of the toxicity or poor palatability, the concentration in the remaining crop/gizzard content would be low, even though the animal was actually poisoned. In addition, the detection of residues in the liver reveals the systemic passage of the product. But, the threshold of imidacloprid concentration in the liver above which we can conclude with little doubt that imidacloprid poisoning is the cause of death is not clearly defined. In experimental conditions, poisoning is usually associated with imidacloprid concentrations in the liver above 5 mg/kg (Pflüger, personal communication in Berny et al. 1999). However, in experimental conditions quails were used and they were fed only dressed seeds, which may result in higher concentration. In field condition, this concentration is rarely reached (Berny et al. 1999, our results) and a concentration ≥ 1 mg/kg appears more realistic for diagnostic purposes. On these bases, we classified as “strong” (answer R3a, Fig. 1) the pathologic relevance of the incidents for which nervous disorders were described at least for one individual, and the incidents for which an imidacloprid concentration ≥ 1 mg/kg was found in the liver, provided that post-mortem examinations suggested an acute pathologic process. However, the investigations performed during the SAGIR process can lead to search for other substances (if, for example, other substances have been used where and when the incident occurred; see “Necropsy and Residue Analysis” section). Consequently, as clinical signs of pesticide poisoning are rarely specific, if another substance has been detected we systemically classified the pathologic relevance of these incidents as “low” (answer R3b see Fig. 1). In this case, it is difficult to assess which one of substances (if any) is more likely to have caused the death (or the interaction between the two substances).

## Results

### Incidents involving imidacloprid

From the 1 January 1995 to the 31 December 2014, the SAGIR network reported 3130 suspected poisoning incidents, of which 103 associated with a real exposure to imidacloprid. Two incidents were clearly linked with a misuse of imidacloprid (i.e. pile of seeds found outside crop fields) and thus removed from this analysis.

So, 101 incidents consistent with an agricultural use of imidacloprid were reported, totalling ≥734 individuals of at least 11 species of birds and 1 mammal (Table 1). The mean number of individuals per incident was 7 ± 15 (sd) [min 1–max 100]. Clustered incidents constituted 71% of incidents. Among them, 67 monospecies- and 5 multispecies-clustered incidents were described. One multispecies-clustered incident implicated two species of the same family (Phasianidae) and four clustered incidents implicated species of two different families (Phasianidae and Columbidae, Columbidae and Fringillidae, Columbidae and Sturnidae, Columbidae and Laridae).

**Table 1:** Number of incidents (for which residue analyses detected imidacloprid) reported by SAGIR from the 1 January 1995 to the 31 December 2014, and number of dead or dying animals per species

	Number of dead or dying animals	Number of incidents
Birds		
Grey partridge (*Perdix perdix*)	95	38
Feral/rock pigeon (*Columba livia*)	341	21
Pigeon sp.^a^	199	20
Wood pigeon (*Columba palumbus*)	48	11
Partridge sp.^b^	15	4
Red-legged partridge (*Alectoris rufa*)	3	3
Ring-necked pheasant (*Phasianus colchicus*)	2	2
Eurasian collared dove (*Streptopelia decaocto*)	20	1
Stock dove (*Columba oenas*)	4	1
Common crane (*Grus grus*)	2	1
Black-headed Gull (*Chroicocephalus ridibundus*)	1	1
Common starling (*Sturnus vulgaris*)	Not specified (≥1)	1
Fringillidae	Not specified (≥1)	1
Mammals		
Brown hare (*Lepus europaeus*)	2	1

Species are separated by class and ranked in decreasing order of the number of incidents

^a^ *C*. *palumbus*, *C. livia* or *C. oenas* not specified

^b^ *A*. *rufa* or *P*. *perdix* not specified

Pigeons and grey partridges were involved respectively in 51% and 38% of the total incidents, but 81% of the total number of dead or dying animals were pigeons compared to 13% for grey partridge. More pigeons were involved by incident than grey partridges (Mann-Whitney *U* test, *p* value < 0.001).

### Temporal distribution of incidents

**Fig. 2:**
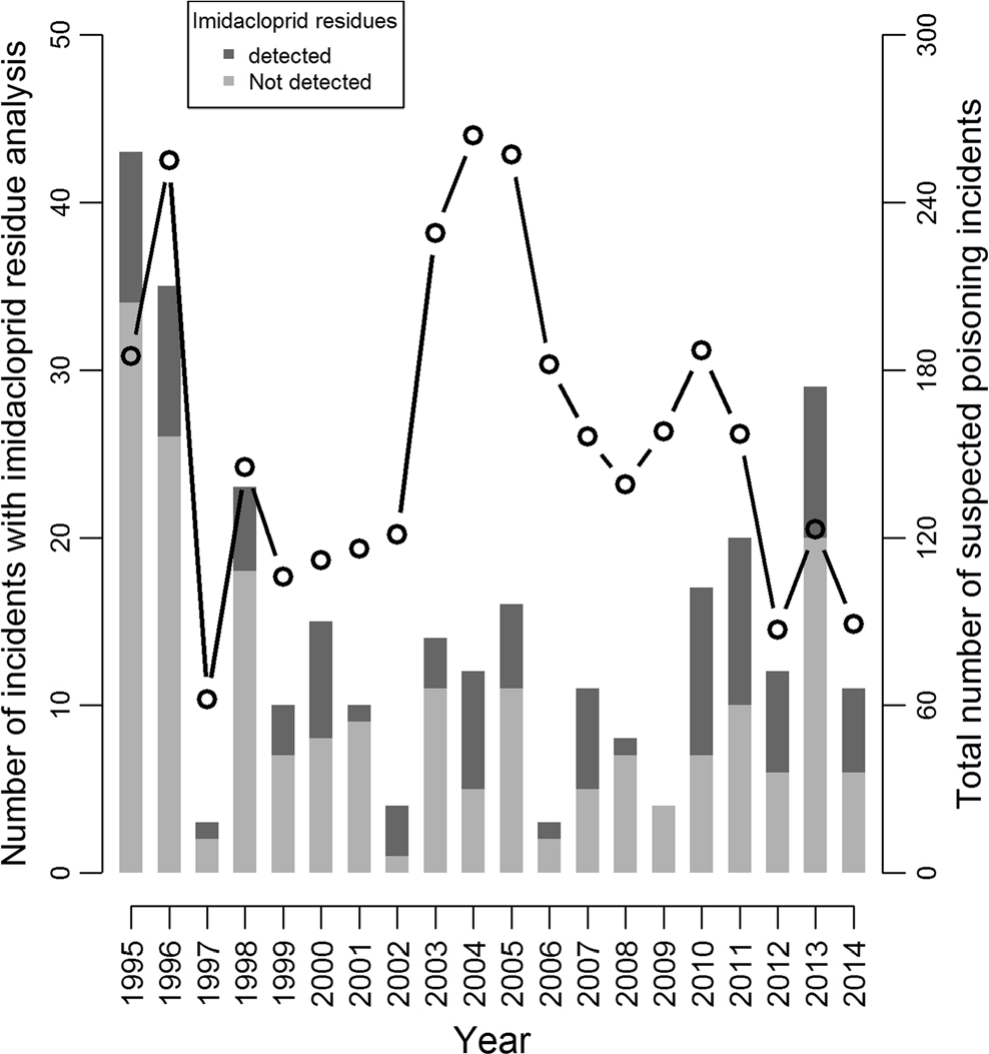
Yearly distribution of incidents for which residue analysis of imidacloprid has been performed (*stacked bars* and *left hand axis*), and total number of suspected poisoning incidents (*curve* and *right hand axis*) reported by SAGIR from the 1 January 1995 to 31 December 2014

Since 1995, incidents were reported all years but one (Fig. 2), numbers varying between 0 (in 2009) and 10 (in 2010 and 2011).

**Fig. 3:**
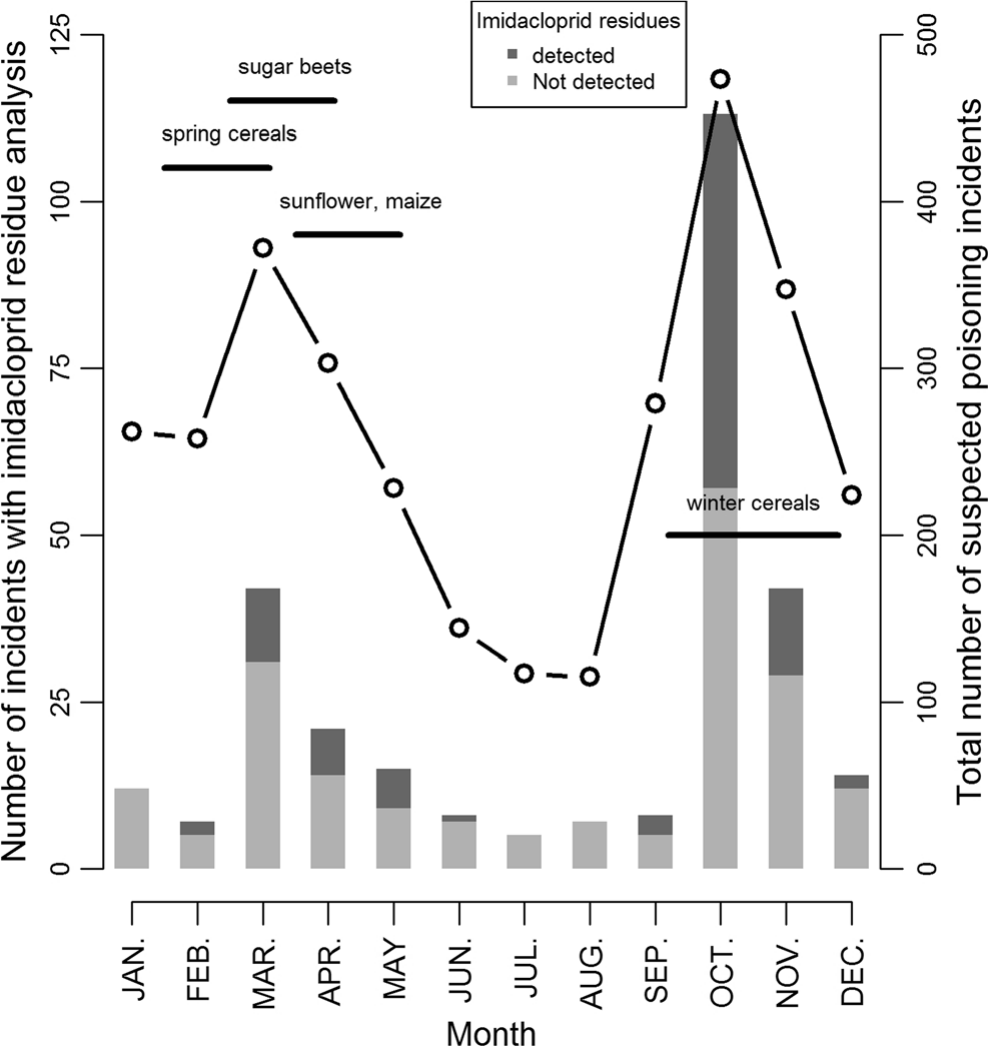
Monthly distribution of incidents for which residue analysis of imidacloprid has been performed (*stacked bars* and *left hand axis*), and total number of suspected poisoning incidents (*curve* and *right hand axis*) reported by SAGIR from 1 January 1995 to 31 December 2014, and corresponding sowing periods of maize, spring and winter cereals, sunflower, and sugar beet

Monthly distribution of imidacloprid-analysed incidents was similar to the monthly distribution of total suspected poisoning incidents. However, imidacloprid-confirmed incidents were more frequently reported in October–November and February–May periods coinciding respectively with the “optimal” sowing period of winter cereals, and spring cereals, sugar beets, sunflower and maize (Fig. 3). For these crops, imidacloprid is or was authorized as seed treatments. Autumn incidents (from September to December) represented 73.3% of the total incidents and they involved more individuals than spring incidents (February to June; Mann-Whitney *U* test, *p* value < 0.01; number of animals (median [min-max]): spring incidents 1 [1–20]/autumn incidents 3 [1–100]).

**Fig. 4:**
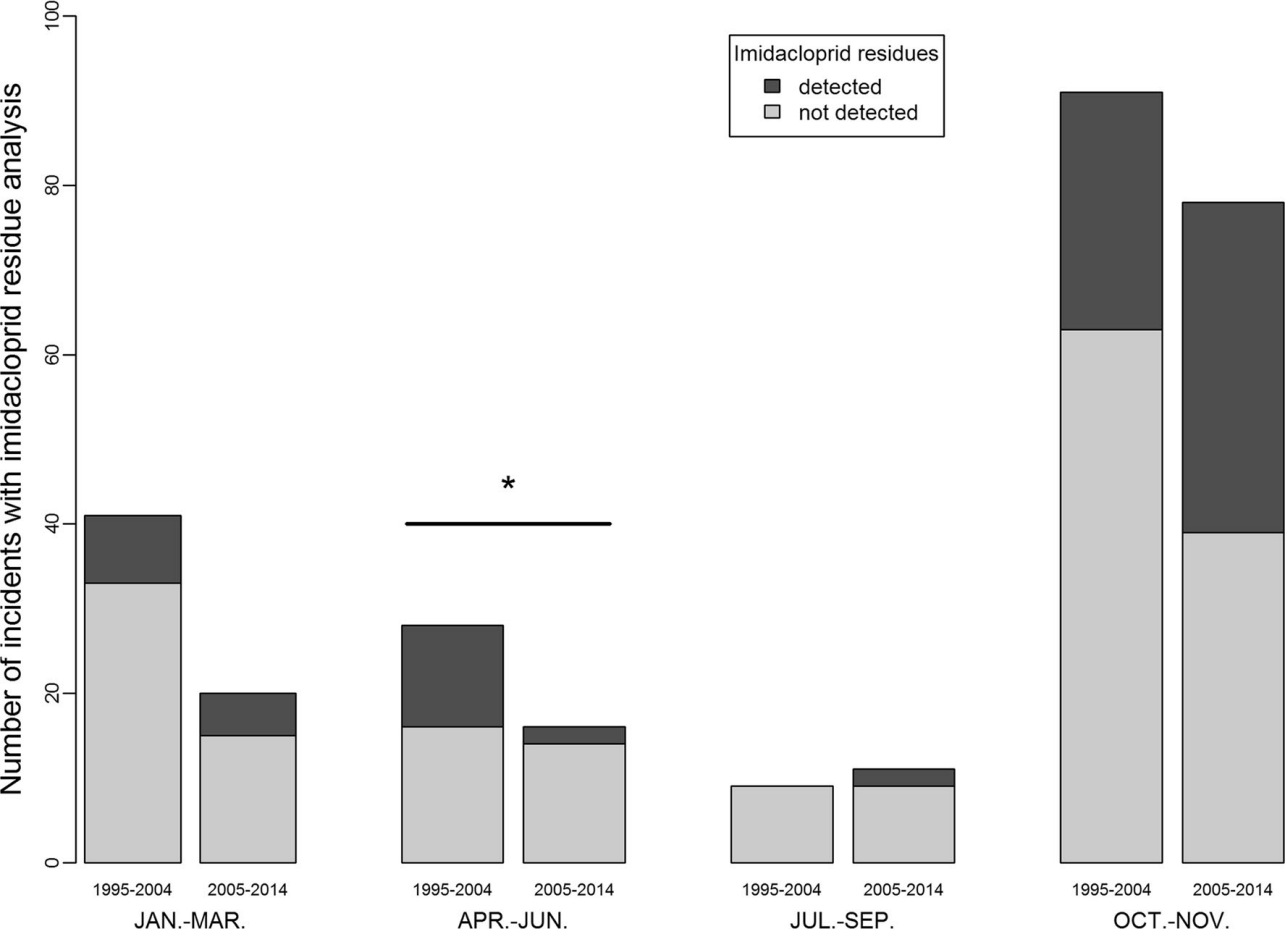
Distribution of incidents for which residue analysis of imidacloprid has been performed reported by SAGIR before (1995–2004) and after (2005–2014) the ban use of imidacloprid as maize seed treatment in 2004; *indicates that the proportion of the number of incidents for which imidacloprid residues have been detected has significantly decreased after 2004 (Fisher’s exact test, *p* < 0.05)

After 2004, the proportion of imidacloprid-confirmed incidents among the total number of imidacloprid-analysed incidents was significantly lower during maize sowing period (Fig. 4). We found this pattern of evolution only for this period (Fig. 4).

### Spatial distribution of incidents

**Fig. 5:**
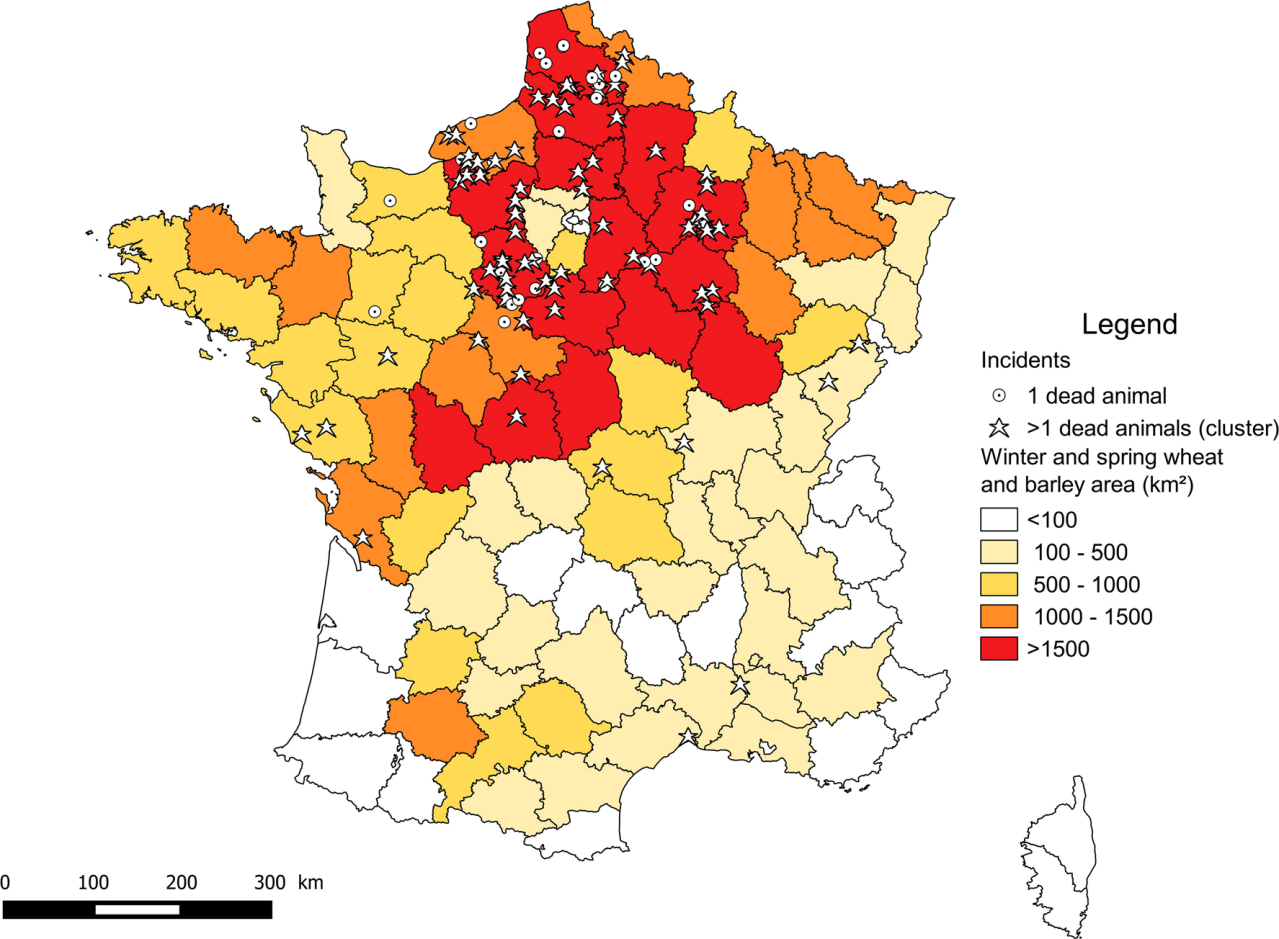
Total acreage (km^2^) of winter and spring wheat and barley in French metropolitan departments (average of yearly acreage from 1995 to 2014, source http://agreste.agriculture.gouv.fr) and geographical location of incidents (for which residue analyses detected imidacloprid) reported by SAGIR from 1 January 1995 to 31 December 2014

**Fig. 6:**
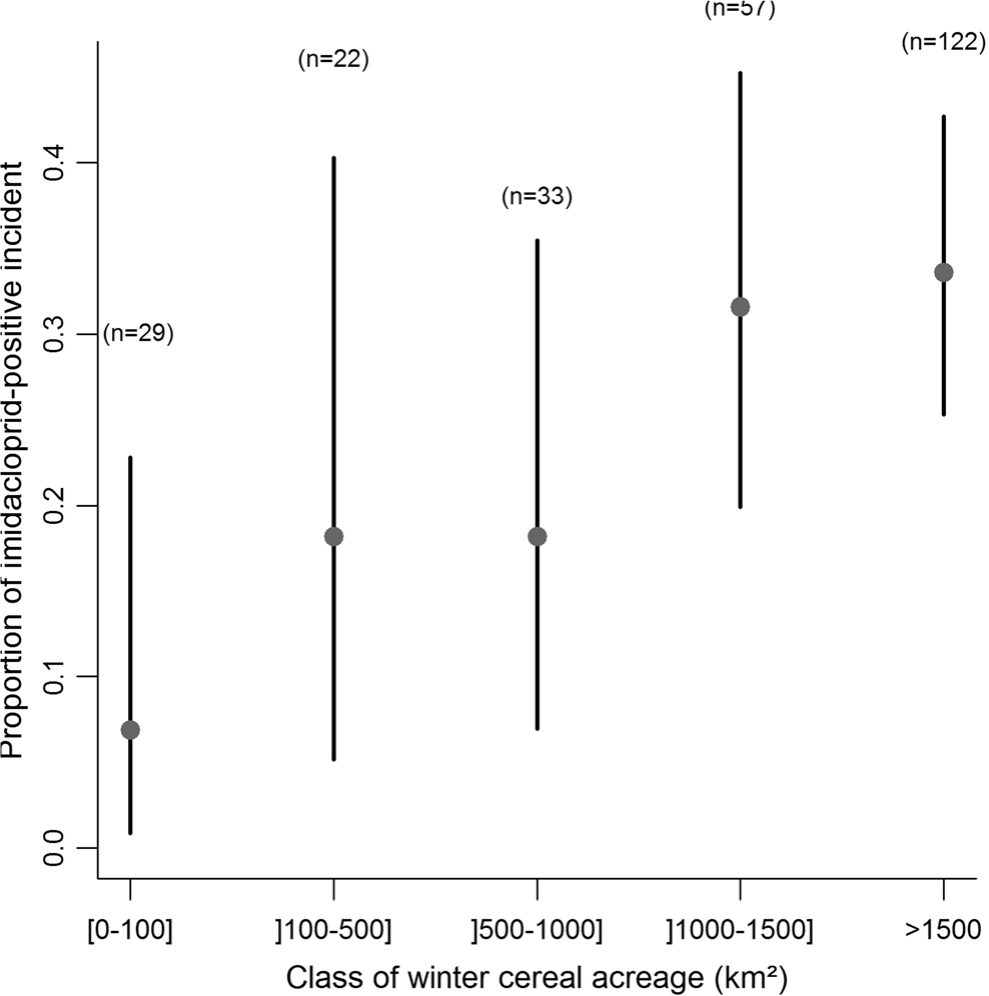
Evolution of the proportion of the number of autumn (from September to December) incidents for which residue analysis detected imidacloprid among the total “partridge” and “pigeon” suspected poisoning incidents reported by the SAGIR from 1 January 1995 to 31 December 2014 according to the class of the winter wheat and barley acreage (average of yearly acreage from 1995 to 2014, source http://agreste.agriculture.gouv.fr) of the French metropolitan department; *Vertical bars* indicate the 95% confidence interval)

Incidents were mostly located in North-Central France, corresponding to intensive cereal growing areas (Fig. 5). We found a tendency to detect a higher proportion of imidacloprid-confirmed incident in autumn in the departments with higher acreage of winter cereals (Fig. 6).

Dead or dying animals were found on or close to recently sown fields in 37.6% of the total incidents. In 28.7% of incidents, animals were found in crops but the plant growth stage was not specified. Other kinds of habitat (woods, fallow, meadow, village, isolated farm) were reported in 23.8% of incidents. For 9.9% of incidents, no information on the discovery site was specified. The habitats where animals were found varied among species categories. No grey partridge incident (*n* = 38) was found in other kinds of habitat than crops compared with 44.2% of pigeon incidents. Seventy-eight percent of pigeon incidents found in other kinds of habitat were reported near human habitation or human activity area (e.g. isolated barn).

### Gross pathology, clinical picture and residue analysis

Nervous disorders (sudden fall in flight, ataxia, paralysis or paresis, behaviour disturbance, disorientation, impaired alertness, apathy) were described in 39.6% of incidents (*n* = 101). Observation of such effects did not differ significantly between incidents involving grey partridges (36.8%, *n* = 38) and those involving pigeons (44.2%, *n* = 52). For one incident, disorientation was associated with red droppings. Eleven birds involved in four incidents showed reversible nervous disorders and were released safe after a period of captivity ranging from 4 h to 2 days.

Dressed seeds were reported in digestive contents for 54% of the 26 incidents in February–May and 71% of the 69 incidents in October–November. Dressed seeds were cereals (both barley and wheat), maize and beet respectively in 50, 5 and 1 cases. In ten cases, the kind of seeds was not specified. Sunflower dressed seeds have never been reported.

Two hundred thirty-nine animals were collected totalling approximately one-third of the total number of dead animals reported. We reviewed post-mortem lesions observed on grey partridges (*n* = 82) and pigeons (*n* = 128) totalling approximately 88% of the total number of animals collected. 90.2% of grey partridges and 64.8% of pigeons were reported in good body condition, suggesting an acute process (Table 2). Signs of predation were more frequently noticed for grey partridges (31.7%) than pigeons (0.8%) (Table 2) but we cannot determine if they were *ante*- or *post-mortem*. Post-mortem examinations showed either no lesion or non specific lesion such as congestion or haemorrhage of the lung, the liver, the kidney, and the intestinal tract, discolouration of hepatic parenchyma (Table 2). Lesions, especially congestion lesions, were more frequently reported in grey partridges than pigeons but besides these slight differences, the lesions found in grey partridges and pigeons are very similar (Table 2).

**Table 2:** Results of post-mortem examination of grey partridge (*Perdix perdix*) and pigeons (*Columba* *palumbus,* *Columba* *livia* and *Columba oenas*) carcasses

	Grey partridge	Pigeons	Statistical differences^d^
Total number of individuals	82	128	
Body condition (n)			
Good	74	83	ns
Bad (cachexia)	0	2	
Not specified	8	43	
Signs of predation (n)			
No	53	116	*
Yes	26	1	
Not specified	3	11	
Summary of lesions (n)			
No lesion	29	57	*
Congestion^a^	29	17	*
*Intestinal* *tract*	*6*	*14*	
*Liver*	*11*	*6*	
*Kidney*	*11*	*3*	
*Lung*	*11*	*5*	
Haemorrhage^a^	21	29	ns
*Intestinal tract*	*8*	*17*	
*Liver*	*5*	*2*	
*Kidney*	*4*	*1*	
*Lung*	*14*	*18*	
Other^b^	6	3	ns
*Discolouration of the hepatic parenchyma*	*6*	*2*	
*Liver necrosis*	*0*	*1*	
Inconclusive post-mortem examination^c^	9	30	

^a^Affected organs are indicated below in italics

^b^Observed lesions are specified below in italics

^c^State of carcass (e.g. beginning of putrefaction process, lack of some organs) did not allow complete post-mortem examinations or results not reported in the SAGIR database

^d^Difference between grey partridge and pigeons were investigated with Fisher’s exact tests, *significant statistical differences (*p* < 0.05), *ns* no significant statistical differences

Bacteriological and/or parasitological examinations were carried out in 99 carcasses. A few bacteria or parasites were isolated, but they were not considered as relevant for diagnosis in all cases.

We performed residue analyses on 118 single individuals and 33 analyses on a pool of different animals (representing 114 different animals) (see Online Resource for details). Analyses were performed only on crop/gizzard in 52% of cases, on crop/gizzard and the liver in 42%, and only on the liver in 5%. For one case, analysis was performed on a sample of seeds found close to the carcass, and in two cases, the matrix was not specified (see Online Resource for details).

**Fig. 7:**
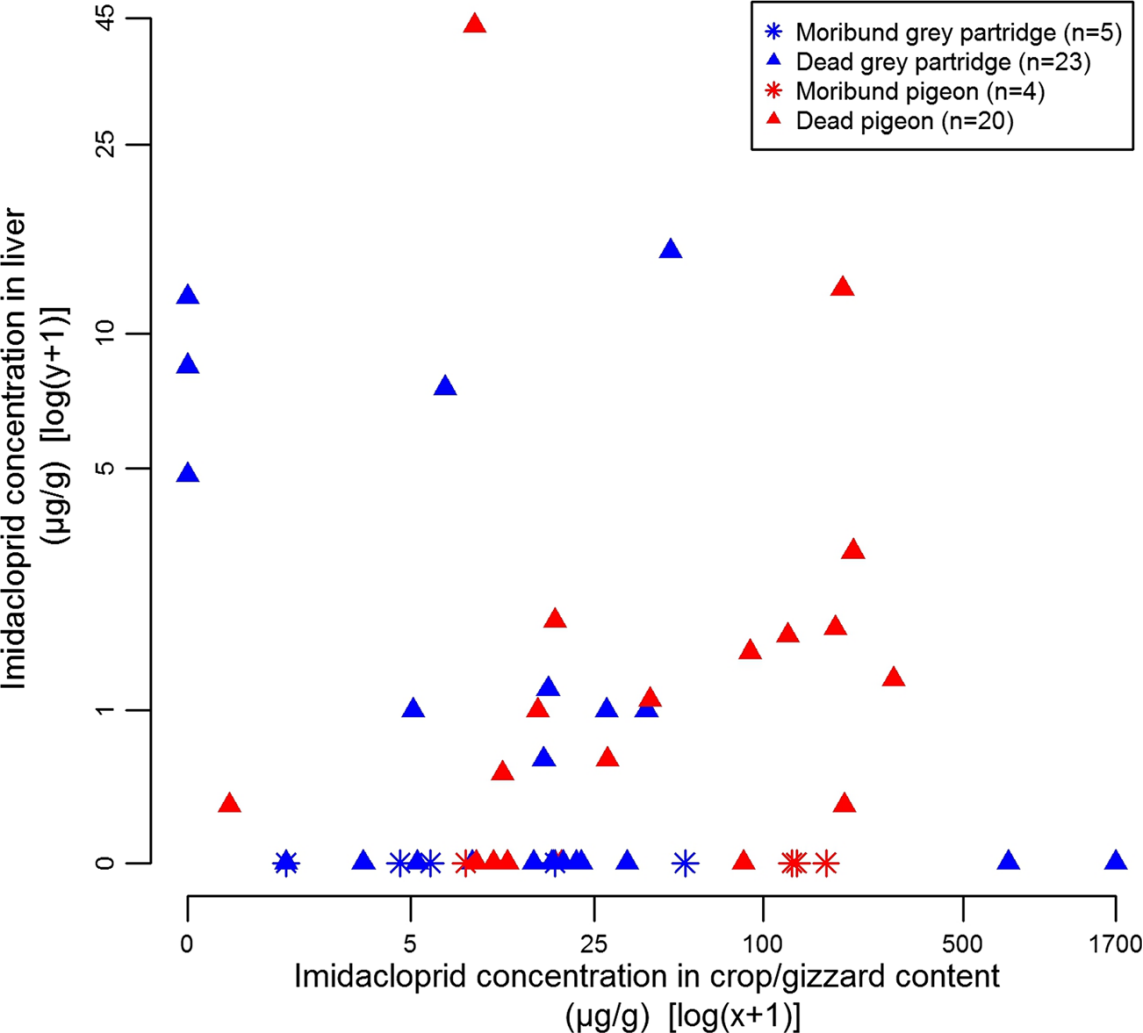
Evolution of the imidacloprid concentrations quantified in the liver and crop/gizzard of grey partridges (*Perdix perdix*) and pigeons (*Columba palumbus*, *Columba livia* and *Columba oenas*) according to that they were found dead or moribund (results of analyses performed separately on individuals)

Hereafter, we focused on individual residue analyses in crop/gizzard and liver of grey partridges (*n* = 46) and pigeons (*n* = 57), totalling approximately 90% of the total number of individual analyses. We found a significant difference between crop/gizzard concentration of imidacloprid in partridges and pigeons (Mann-Withney *U* test, *p* < 0.05) but not for liver concentration (Table 3). Nevertheless, for the individuals with both the crop/gizzard and the liver analysed, we found a larger proportion of partridges with imidacloprid residues detected in crop/gizzard but not in the liver (partridge: 72%, *n* = 25; pigeons: 42% *n* = 24; Fisher’s exact test, *p* < 0.05). We found no relation between individual crop/gizzard and liver concentration (Fig. 7). All individuals with imidacloprid residues detected in the liver were found dead (*n* = 26) while 32% of individuals with no imidacloprid residues detected in the liver but in the crop/gizzard (*n* = 28) were found moribund. Overall, in more than half the cases when residue analyses were performed on both the crop/gizzard and the liver, imidacloprid residues were not detected in the liver (all species, individual analyses and pool of individuals, Table 4)

**Table 3:** Levels (median and range) of imidacloprid measured in the crop/gizzard content and the liver of grey partridges (*Perdix* *perdix*) and pigeons (*Columba palumbus*, *Columba* *livia* and* Columba* *oenas*)

	Crop/gizzard			Liver		
	Number of analyses	Number of positive^a^ results	Median (min–max)^b^	Number of analyses	Number of positive^a^ results	Median (min–max)^b^
Grey partridge	46	43 ^c^	15.0 (0.9–1706.0)	28	10 ^d^	3 (0.6–15.0)
Pigeons	51	51	34.7 (0.4–286.7)	29	16 ^d^	1.4 (0.3–43.5)

^a^i.e. above the detection limit

^b^Calculated only from positive analyses. Values in microgram per gram

^c^Imidacloprid residues were found in the liver of animals for the three cases below the detection limit

^d^For all individuals for which liver analyses were below the detection limit, imidacloprid residues were found in crop/gizzard, except three pigeons for which crop/gizzard could not be analysed but for which imidacloprid was detected in other individuals from the same incident

**Table 4:** Detail of matrix (crop/gizzard and the liver) in which imidacloprid was detected when analyses were performed in both crop/gizzard and the liver

	Imidacloprid residues detected		
	in crop/gizzard and the liver	only in crop/gizzard	only in liver
Individual (n)	22	29	3
Pool of individuals (n)	4	4	0
%	42	53	5

### Diagnosis of poisoning by imidacloprid-treated seeds in the context of an agricultural use

#### Question 1: exposure assessment

Since we only took into account incidents for which imidacloprid has been detected, exposure was classified as “proved” (answer R1a, see Fig. 1) for all but three incidents. For these incidents exposure was classified as “strongly suspected” (answer R1b, see Fig. 1). In two cases, the matrix in which imidacloprid residues were detected was not known and in one case, residue analysis was performed only on seeds found close to the carcass. For all the other incidents, imidacloprid residues were detected in crop/gizzard and/or the liver (see Online Resource for details).

#### Question 2: assessment of epidemiological credibility and relevance

Almost all incidents were detected during the sowing seasons of crops for which imidacloprid is (or was) authorized as seed treatment in France (Fig. 3). All incidents were detected in areas where these crops were cultivated. The epidemic curve of all clustered incidents is consistent with poisoning (all individuals found dead the same day and carcasses separated by less than a few meters). However, we classified the epidemiological credibility as “low” (answer R2b, see Fig. 1) for 26 clustered incidents. These incidents were found outside crops (e.g. village, isolated farm), and we could not totally exclude that animals had fed on dressed seeds which would not be drilled, as for example an open bag of dressed seeds in a barn. For the 46 other clustered incidents, the epidemiological credibility was classified as “strong” (answer R2a, see Fig. 1). The remaining 23 incidents were single incidents and, thus, they were classified as “low” for epidemiological relevance (see Online Resource for details).

#### Question 3: assessment of pathologic relevance

No post-mortem examinations clearly pointed out anything else other than acute pathologic process.

Other residue analyses were carried out for 47 incidents. Other substances were detected for 13 of them, thus these incidents were classified as “low” for pathologic relevance. Forty-seven incidents were classified as “strong” for the pathologic relevance because nervous disorders have been observed and/or the liver imidacloprid concentration has been equal or higher than 1 mg/kg for almost one individual of the incident. The remaining 41 incidents were classified as “low” for the pathologic relevance (see Online Resource for details).

As a result, according to this classification approach (above and Fig. 1), the diagnosis of poisoning by imidacloprid-treated seeds in the agricultural context was classified as “very likely”, “likely” and “possible” for 21%, 49% and 30% of the incidents, respectively (see Online Resource for details).

## Discussion

Since 1995, incidents have been detected almost every year and related to imidacloprid-treated seeds of different crops with different sowing periods. Furthermore, mortality due to poisoning by treated seeds was ranked as at least “likely” in 70% of incidents. Various environmental and anthropogenic factors affect the collection of wildlife mortality data (e.g. Berny 2007; de Snoo et al. 1999; Vyas 1999; and below). As a result, it is well acknowledged that the actual number of wildlife mortality largely exceeds the number of carcasses actually recovered (Vyas 1999). Consequently, the first major contribution of this article is to confirm that in real conditions granivorous birds are regularly exposed to imidacloprid-treated seeds that can result in acute lethal or sublethal effects. This work also shows that the two main factors (seed burying and avoidance of treated seeds) supposed to mitigate the risk for granivorous birds are not completely efficient in natura.

### Efficacy of mitigation measures

#### Seed burying

For the incidents reported by the SAGIR, we are not able to estimate to what extent these incidents may be attributed to the noncompliance of use instructions (e.g. spillage not removed). The presence of piles of spilled seeds has been reported in very few incidents. However, a precise assessment of the amount of seed available at the soil surface of sown fields around the incident discovery sites was not systematically performed. In addition, distinguishing between a clear noncompliance of good agricultural practices and an actual technical inability for farmer to strictly follow the use conditions may be sometimes tenuous.

In France, to protect wild birds and mammals, label instructions are to both remove any spilled seeds and incorporate all dressed seeds into the soil. Yet, in routine use, seed burying is rarely 100% effective, especially on the headland (i.e. the place where the drilling implement is turned when sowing) and various factors affect the proportion of seeds actually buried (e.g. Pascual et al. 1999a, b; de Snoo and Luttik 2004). For example, without taking spillage into account, de Snoo and Luttik (2004) found that 9.2% of drilled winter wheat seeds remain on the soil surface on the headland. In France, the winter wheat seeding rate varies from 150 seeds/m^2^ (in both best soil condition and sowing date) to 450 seeds/m^2^ (in both worst soil condition and sowing date). So, according to de Snoo and Luttik (2004)‘s results, between 14 and 41 seeds/m^2^ would remain on the soil surface. These estimations are consistent with our own results. We estimated that an average of 8 to 96 seeds/m^2^ remained on the soil surface on the headland of 15 winter wheat and barley fields in autumn 2013 (unpublished data). Winter wheat seeds are treated with 0.7 g of imidacloprid per kilogramme of seeds (https://ephy.anses.fr/). With a thousand grain weight of wheat seeds ranging from 40 to 60 g, one seed is thus treated with 0.028–0.042 mg of imidacloprid. Consequently, for a grey partridge weighing approximately 390 g the median lethal dose of 13.9 mg/kg (Gibbons et al. 2015) is reached with the ingestion of between 129 and 194 wheat seeds. For a feral pigeon (*C*. *livia*) weighing approximately 300 g (http://app.bto.org/birdfacts/results/bob6650.htm), the median lethal dose of 25 mg/kg (Gibbons et al. 2015) is reached with the ingestion of between 179 and 268 seeds. So, according to de Snoo and Luttik (2004)‘s estimates, these amounts would be found on the soil surface of areas ranging from approximately 3 to 14 m^2^ for grey partridges and 4 to 19 m^2^ for pigeons. As a consequence, the amount of seeds that would remain on the soil surface in a routine use would be high enough to cause mortality.

However, according to the optimal foraging theory, areas with spillage (i.e. local high seed density) may be more attractive for birds than areas with only irregularly scattered seeds (de Leeuw et al. 1995). Nevertheless, Murton et al. (1963) found that only food densities <2 grains/m^2^ were too low for the wood pigeon to exploit successfully. Thus, even without spillage spots, densities of grains in cereal fields in autumn are sufficient to attract wood pigeons. On the contrary, Moorcroft et al. (2002) found that autumn grey partridges rarely feed on stubble fields where cereal grain density was <50 seeds/m^2^. For grey partridge, thus, fields with only scattered seeds may have only a low food value. Nevertheless, some other factors such as the density of other seeds in sown fields (e.g. weed seeds) or the availability of other fields with higher food values may affect the attractiveness of autumn cereal sowing. Furthermore, even when seeds are completely buried into the soil, bird exposure can still occur. For instance, skylarks bring seeds to the surface by uprooting seedlings (Green 1978).

#### Avoidance of Imidacloprid-treated seeds

Imidacloprid-treated seed avoidance, observed in captive birds, is due to a conditioned aversion mediated by sickness. This aversion occurs after a first experience of treated seeds ingestion (Avery et al. 1993; Lopez-Antia et al. 2014). The efficiency of this kind of learned avoidance requires that sublethal effects leading to rejection occurred well before a lethal dose is ingested. The amount of toxic needed to cause rejection (in relation to lethal dose) and the speed at which it occurs, as well as the feeding rate of birds are, thus, important parameters. Yet, various factors that may happen in the wild, as starvation (Pascual et al. 1999c), predation risk (Avery et al. 1994), availability and unpredictability of alternative food (Lopez-Antia et al. 2014; Murton and Visozo 1963), or competition (Mckay et al. 1999) may alter feeding behaviour to such an extent that lethal doses of pesticide could be ingested before avoidance occurs, or even overcome avoidance.

Furthermore, the amount of ingested imidacloprid-treated seeds can widely vary among individuals. As a result, even in “optimum” captivity conditions (i.e. availability of alternative food and without food shortage) the avoidance of imidacloprid did not prevent the occurrence of nervous disorders (Avery et al. 1993) or death (Lopez-Antia et al. 2014). Although the observed nervous disorders are transitory, in the field they could cause the death of wild birds for example by making them more vulnerable to predation or collision with vehicles, as well as favouring falling in flight. Such associated incidents could explain the haemorrhagic lesions observed in many birds. These secondary effects may also suggest a higher risk for birds in nature, in relation to indirect mortality.

Another study (Soyez 1998 in ANSES 2011) showed the variable nature of avoidance of imidacloprid-treated seeds that may be reduced when seeds are leached by rain or “aged” a few hours before being accessible for captive birds. This decrease of repellent effect could result from the dissipation of imidacloprid residues on seeds. Indeed, the concentration of imidacloprid in seeds affects the bird avoidance response (Avery et al. 1993, 1994).

The density of treated seeds at the soil surface may also influence the avoidance response of birds by modifying their rate of seed consumption. The intake rate of grains by birds increases with the density available on the soil surface (Baker et al. 2010; Murton et al. 1963). For example, the seed intake rate varies from about 4 peck/min at low seed densities (2 seeds/m^2^) to about 30 pecks/min at high densities (>150 seeds/m^2^) for grey partridges (Baker et al. 2010), and from about 30 seeds/min at low densities (<20 seeds/m^2^) to about 60 seeds/min at high densities (200 seeds/m^2^) for wood pigeons (Murton et al. 1963). Thus, in a high seed density situation a lethal amount of treated seeds could be ingested before post-ingestional distress happen. As a result, spots of spilled seeds pose a very high risk for granivorous birds.

### Variability in risk factors, incident detection and reporting

The majority of incidents were reported in autumn. Grey partridge and pigeons—especially feral pigeon and to a lesser extent wood pigeon—are the major bird species reported in incidents. These results may reflect specific features in the probability of incidents being detected and reported but also in risk factors.

#### Detection and reporting

Carcass density and morphology (i.e. size and colour), ground-vegetation composition and structure, as well as the level of human activity where mortalities occurred, affect the probability of carcasses being found (Vyas 1999). In addition, once a dead animal is found, other factors such as public awareness of registration scheme, or the affective value of the species found, can also affect incident reporting. For example, the SAGIR mainly relies on hunters. Hunters (and their dogs) are more likely to find wildlife carcasses than other people as jogger or walker. They are generally well informed of the existence of the reporting scheme but their focus is often limited to game species (Berny 2007).

Thus, given these sources of variability, autumn incidents may have been more detected than spring incidents. In autumn, after crop harvest, cereal-growing areas are mainly composed of stubble, bare ground and recently sown crops while in spring, the vegetation is actively growing. In a farmland, small game hunting period is mainly from mid/end September to November. In addition, more individuals were involved in autumn incident. Similarly, grey partridge and pigeon carcasses may have been more detected and reported. They are relatively large game birds compared to other nongame farmland birds such as small passerines. Pigeon incidents may have been even more detected than partridge ones since they involved more individuals and they were detected more frequently close to human activity area (e.g. village, barn, farm). In addition, smaller birds are scavenged more quickly and at a higher proportion than larger birds (Ponce et al. 2010). Thus, the fact that no imidacloprid incidents involving smaller farmland birds were reported in the SAGIR does not mean that they do not occur in the field. For instance, Emberizidae, Fringillidae, Passeridae and Paridae species represent only about 5% of the approximately 15,000 bird data reported in the SAGIR database. Fringillidae mortality was specified in one incident also involving pigeons. Unfortunately, no post-mortem examination and residue analyses could be done on carcasses of this species.

#### Is the exposure to treated seeds higher for some crops?

The majority of incidents were related to winter cereals. Yet, with a mean of 0.035 mg of imidacloprid by seed, cereals are not the most hazardous seeds compared to beet or maize seeds that are treated with 0.9 mg and ~1 mg of active ingredient per seed, respectively (Goulson 2013). So, some factors may have increased the bird exposure to imidacloprid-treated cereal seeds sown in autumn.

First, in France approximately 6.5 millions of hectares of winter cereals are cultivated compared to 2.5 millions of hectares of spring crops (spring cereals, beets, maize, and sunflower) for which imidacloprid is/was used as seed treatment. Besides, successive use restrictions of imidacloprid as seed treatments of different spring crops have occurred since 1995 (sunflower in 1999, maize in 2004, spring cereals in 2014). Thus, although we do not know the yearly share of each crop with imidacloprid seed dressing, the acreage of field sown with imidacloprid-treated seeds was certainly much larger in autumn than in spring.

Second, the proportion of seeds remaining on the soil surface is higher for cereals sown in autumn than in spring, probably due to the unfavourable soil conditions in autumn, and for crops sown with standard drill (as cereals) than crops sown with precision drill (as sugar beet, maize and, sunflower; de Snoo and Luttik 2004). For instance, de Snoo and Luttik (2004) found an average of 0.03 seeds/m^2^ on precision-drilled crops (maize, onion, and sugar beet), that is far below the 2 grains/m^2^ for wood pigeons to exploit these fields. As a result, precision-drilled crop fields may be not attractive to birds due to too low densities of surface grain. Birds are thus probably more exposed to treated seeds of autumn sown cereals than to seeds of spring sown crops.

In addition, other factors as bird preference for some seeds may also influence the degree of exposure to treated seed according to crop. For example, pelleted sugar beet seeds are poorly attractive (Prosser and Hart 2005).

#### Variability of species sensitivity

Grey partridges and pigeons are particularly sensitive to imidacloprid. Indeed, according to the USEPA classification, imidacloprid is highly acutely toxic for both grey partridge and feral pigeon while it is moderately toxic for Mallard (Gibbons et al. 2015). Feeding habits of these species may also increase their exposure to imidacloprid-treated seeds. Pigeons feed on crop sowings, especially cereals (Inglis et al. 1990; M’Kay et al. 1999; Murton and Westwood 1966; Murton et al. 1963), all the more when other preferred food sites, as stubble, are scarce (Inglis et al. 1990; Murton and Vizoso 1963). In autumn, cereal grains represent about 50% of the diet of the grey partridge (Birkan and Jacob 1988) and partridges prefer to forage in field edges. Thus, they will probably be exposed to a higher number of imidacloprid-treated seeds due to the higher number of surface seeds on the headland. Furthermore, the grey partridge and the pigeons store food in their crop from the mid/end of day for digestion during the night (Murton et al. 1963; Nikiforov 1992; Rashotte et al. 1997), but, to the best of our knowledge, whether and how this feeding behaviour could affect the avoidance response has never been studied.

On the other side, smaller sized birds are more exposed to pesticides due to higher daily energy expenditure. As a result, the risk of imidacloprid-treated seed poisoning could be greater for them. Mineau and Palmer (2013) estimated that the ingestion of less than four imidacloprid-treated wheat seeds would have a 50% probability of killing a bird weighing 15 g. Given the number of imidacloprid incidents involving grey partridge and pigeon reported here, and the number of wheat seeds sufficient to reach the LD50 for both the bird species, we can claim that this toxic amount of seeds (for small birds) is commonly and largely available at the soil surface of wheat sown fields. This is also supported by our estimation of surface seeds in fields. However, it is acknowledged that some birds dehusk seeds and that this behaviour is mainly observed in small species (body weight < 50 g) and chiefly in the specialized granivores (finches, sparrows and buntings) (Avery et al. 1997; Prosser and Hart 2005), Thus, the ingestion of imidacloprid together with the consumption of treated seeds may be reduced by this behaviour for small granivorous farmland birds.

### Residue analysis and diagnosis of poisoning

Overall, in more than half the cases when residue analyses were performed on both crop/gizzard and liver the systemic absorption of imidacloprid was not confirmed since imidacloprid residues were not detected in the liver. This may reflect the occurrence of indirect mortalities (e.g. traumatic death due to a falling in flight, as can be suspected from the detection of haemorrhages in dead birds) caused by imidacloprid sublethal poisoning. This assumption is supported by the fact that we detected no imidacloprid residues in the liver of the nine individuals found moribund for which residue analyses were performed in both crop/gizzard content and the liver.

However, Lopez-Antia et al. (2015) found mean concentrations of imidacloprid of 55.3 μg/g and 82.6 ng/g respectively in crop and the liver of 19 dead red-legged partridges exclusively exposed to wheat seeds treated at recommended application rate (0.7 mg of imidacloprid/g of seeds) for 25 days. Their crop concentrations are similar to ours while their liver concentrations are just below our detection limit in the liver (i.e. 100 ng/g). These results suggest that our nondetection of imidacloprid in the liver of some individuals could be sometimes due to a lack of sensitivity in our analytical method rather than an actual absence of imidacloprid residues. Their findings also indicate that mortalities can be associated with very low imidacloprid liver levels.

In very few cases, we observed a great variability in imidacloprid concentration (crop/gizzard and liver) among individuals of the same clustered incident (see Online Resource). This could be explained by the great individual variability of treated seed consumption, observed in avoidance studies. Furthermore, we found no relation between crop/gizzard concentration and liver concentration. Factor as regurgitation might have been affected the results of crop/gizzard content analyses, but does not necessarily prevent mortality (Pascual et al. 1999d). Further works are required to better understand the relation and variation in both crop/gizzard and liver imidacloprid concentration and, thus, to improve the diagnosis of imidacloprid poisoning.

### Impact of imidacloprid poisoning on bird populations

How the imidacloprid-related mortalities affect farmland bird populations is not known yet. The impact at the population scale is likely to depend upon the status of the population and the timing of mortality.

First, the rate of mortality attributable to imidacloprid poisoning in different bird populations is still unknown. Second, compensatory mortality or natality (see for instance Boyce et al. 1999) may mitigate the effects of these mortalities on populations. Density-dependent overwinter survival and/or natality were found, for example, in grey partridge (e.g, Bro et al. 2003; Panek 1997; Rotella et al. 1996) and pigeons (e.g. Hetmański and Barkowska 2007; Kautz and Malecki 1990; Murton et al. 1974). Seasonal timing in these anthropogenic mortalities and density dependence are important factors determining the nature of the demographic response (Boyce et al. 1999; Kokko 2001). Anthropogenic mortalities are more likely to be compensated for when mortalities are time-limited and happen before a seasonal density-dependent mechanism. Thus, with respect to density-dependent overwinter survival, autumn imidacloprid incidents are more likely to be compensated for than spring incidents. In addition, spring incidents occur principally in the late winter/early spring, at the early beginning of reproduction season of the majority of farmland bird species. Additive mortality is indeed more likely at this stage of the year, and spring pesticide exposure may potentially impact the reproductive success.

The bird population status is another relevant factor that underlies the demographic response to these anthropogenic mortalities. Mortality is more likely to be additive when populations are in decline or at low-density (e.g. Bartmann et al. 1992). In France, short-term (2001–2012) breeding population trends are considered to be fluctuating for both grey partridge and stock dove, increasing for wood pigeon and unknown for feral/rock pigeon (Comolet-Tirman et al. 2015). Thus, these imidacloprid casualties may have limited effects on at least wood pigeon, grey partridge and stock dove global population. However, many other farmland bird populations still decline in France (Comolet-Tirman et al. 2015) and Europe (EBCC 2015). Thus, even though imidacloprid-related casualties are probably not the primary cause of this continuing decline, under some circumstances, they could be an aggravating factor.

Moreover, imidacloprid poisoning of juveniles of some bird species may be a possibility, depending upon the timing of hatching and sowing, and the diet of chicks (while the fledglings of many granivorous birds are mostly insectivore especially in the first weeks of their life). If it occurred, this type of incidents would go totally undetected in the SAGIR Network.

## Conclusion

Given the different sources of variability of the rate of seed burying and the avoidance behaviour to imidacloprid-treated seed, we may argue that occasional imidacloprid poisoning is expected, especially for more sensitive species and when good agricultural practices and use instructions are not followed. But given the regular detection of incidents over the years and the opportunistic *modus operandi* of the SAGIR, we can clearly wonder about both the actual effectiveness of these mitigation factors and the actual impact of direct effects of imidacloprid on bird populations. All the more so that, whether or not these mortality events are due to the noncompliance of good agricultural practices, they appear to be the result of common usual practices since we found a tendency to detect a higher proportion of imidacloprid-confirmed autumn incidents in departments with higher acreage of winter cereals. However, this late point should deserve more thorough analysis.

As a conclusion, all these findings call to reconsider the impact of imidacloprid on farmland bird populations, especially when it is used as seed treatment of winter cereals. The actual efficiency of factors supposed to reduce the exposure of birds in the field should be checked more thoroughly before taking them into account in risk assessment. In addition, refinement of the risk assessment should be more realistic using a more global approach (acute toxicity, sublethal effects, reproductive effects, food reduction) and should distinguish several species to take their diet, energetic, behavioural specificities into account.

## Electronic supplementary material

ESM 1 (PDF 80 kb)
