# Supplementary material for: Field evidence of bird poisonings by imidacloprid-treated seeds: a review of incidents reported by the French SAGIR network from 1995 to 2014
Source: Environ Sci Pollut Res Int. 2016 Dec 27;24(6):5469–85. doi: 10.1007/s11356-016-8272-y (PMC5352772; doi:10.1007/s11356-016-8272-y)
Supplement: Supplementary file 1 — (PDF 80 kb) [file 11356_2016_8272_MOESM1_ESM.pdf]

Online Resource. Detail of incidents reported by the French SAGIR Network from the 1st January 1995 to the 31th December 2014 for which toxicological analyses detected imidacloprid residues (All the concentrations are given in mg/kg; "< LoD" means below the limit of detection; "positive" means that residues were detected but concentrations were not reported ; a: Black-headed Gull (*Chroicocephalus ridibundus*), Brown hare (*Lepus europaeus*), Common crane (*Grus grus*), Common starling (*Sturnus vulgaris*), Eurasian collared dove (*Streptopelia decaocto*), Feral/rock pigeon (*Columba livia*), Grey partridge (*Perdix perdix*), Partridge sp. (*Alectoris rufa* or *Perdix perdix* not specified), Pigeon sp.(*Columba palumbus*, *C. livia* or, *C. oenas* not specified), Red-legged partridge (*Alectoris rufa*), Ring-necked pheasant (*Phasianus colchicus*), Stock dove (*Columba oenas*), Wood-pigeon (*Columba palumbus*) ; b: When the same matrix were not analysed for all the individuals of the same incident number of analysed animals for each category of matrix is given in brackets, for the 2 cases "matrix not specified" and the case "seeds" detected concentration is given in brackets ; c: When residue analysis results are the same for several animals the number of individuals is given in brackets).

Year	Month	Cluster	Species[a]	Number of dead animals	Kind of habitat at the discovery site	Nervous disorders	Number of collected animals	Dressed seeds in crop/gizzard content (type of seeds)	Type of residue analyses	Number of animals analysed for imidacloprid residues	Analysed matrix[b]	Imidacloprid concentration in Crop/gizzard content[c]	Imidacloprid concentration in liver[c]	Other searched substances	Other active substance detected (analysed matrix; detected concentration)	Responses to the diagnostic approach to estimate the degree of certainty that mortalities are due to imidacloprid-treated seeds poisoning and degree of certitude (see Fig 1. and "Results" section)			
																Question 1 Exposure assessment	Question 2 Epidemiological relevance and credibility?	Question 3 pathologic relevance ?	Degree of Certitude
1995	October	No	Ring-necked pheasant	1	Crops (plant growth stage not specified)	Yes (fall in flight)	1	Yes (Not specified)	Individual	1	Crop/gizzard	10.3	-	No	-	a : Proved (imidacloprid residues detected in digestive tract)	b : Low (Single animal incident)	a : Strong (nervous disorders observed)	Likely
1995	October	Yes	Grey partridge	4	Crops (sowing)	No	4	Yes (Not specified)	Individual	4	Crop/gizzard	2.2 ; 4.5 ; 4.4 ; 6.1	-	No	-	a : Proved (imidacloprid residues detected in digestive tract)	a : Strong (Cluster found in crops)	b : Low (Neither nervous disorders observed nor liver imidacloprid residues detected)	Likely
1995	October	No	Grey partridge	1	Crops (sowing)	Yes (ataxia)	1	Yes (Cereals)	Individual	1	Crop/gizzard	26.6	-	Yes (anticoagulants)	No	a : Proved (imidacloprid residues detected in digestive tract)	b : Low (Single animal incident)	a : Strong (nervous disorders observed)	Likely
1995	October	Yes	Grey partridge	3	Crops (sowing)	No	3	Yes (Cereals)	Individual	3	Crop/gizzard & liver	5.1 ; 27.8 ; 38.6	1.0 ; 1.0 ; 1.0	No	-	a : Proved (imidacloprid residues detected in digestive tract and liver)	a : Strong (Cluster found in crops)	a : Strong (imidacloprid concentration in liver ≥ 1mg/kg)	Very likely
1995	October	No	Red-legged partridge	1	Crops (plant growth stage not specified)	Yes (nervous disorders)	1	No	Individual	1	Crop/gizzard	2.5	-	Yes (acetylcholinesterase inhibitors)	No	a : Proved (imidacloprid residues detected in digestive tract)	b : Low (Single animal incident)	a : Strong (nervous disorders observed)	Likely
1995	October	No	Grey partridge	1	Crops (plant growth stage not specified)	No	1	No	Individual	1	Crop/gizzard	3.1	-	Yes (acetylcholinesterase inhibitors)	No	a : Proved (imidacloprid residues detected in digestive tract)	b : Low (Single animal incident)	b : Low (Neither nervous disorders observed nor liver imidacloprid residues detected)	Possible
1995	October	Yes	Grey partridge	3	Crops (plant growth stage not specified)	No	3	Yes (Cereals)	Individual	1	Crop/gizzard	5.8	-	No	-	a : Proved (imidacloprid residues detected in digestive tract)	a : Strong (Cluster found in crops)	b : Low (Neither nervous disorders observed nor liver imidacloprid residues detected)	Likely
1995	November	No	Grey partridge	1	Crops (plant growth stage not specified)	No	1	No	Individual	1	Crop/gizzard	0.9	-	Yes (acetylcholinesterase inhibitors)	No	a : Proved (imidacloprid residues detected in digestive tract)	b : Low (Single animal incident)	b : Low (Neither nervous disorders observed nor liver imidacloprid residues detected)	Possible
1995	November	No	Grey partridge	1	Crops (plant growth stage not specified)	No	1	No	Individual	1	Crop/gizzard	13.2	-	Yes (acetylcholinesterase inhibitors)	No	a : Proved (imidacloprid residues detected in digestive tract)	b : Low (Single animal incident)	b : Low (Neither nervous disorders observed nor liver imidacloprid residues detected)	Possible

1996	March	Yes	Rock/Feral pigeon	10	Other than crops (human activity area)	Yes (paralysis)	1	Yes (Cereals)	Individual	1	Crop/gizzard	0.6	-	Yes (acetylcholinesterase inhibitors)	Acetylcholinesterase inhibitor (Crop/gizzard; positive)	a : Proved (imidacloprid residues detected in digestive tract)	b : Low (Cluster but not found in crop)	b : Low (other pesticides residue detected)	Possible
1996	March	No	Grey partridge	1	Crops (sowing)	Yes (fall in flight)	1	Yes (Cereals)	Individual	1	Crop/gizzard	9.5	-	No	-	a : Proved (imidacloprid residues detected in digestive tract)	b : Low (Single animal incident)	a : Strong (nervous disorders observed)	Likely
1996	April	No	Grey partridge	1	Crops (plant growth stage not specified)	No	1	No	Individual	1	Crop/gizzard & liver	1.2	< LoD	Yes (anticoagulants)	No	a : Proved (imidacloprid residues detected in digestive tract)	b : Low (Single animal incident)	b : Low (Neither nervous disorders observed nor liver imidacloprid residues detected)	Possible
1996	May	Yes	Rock/Feral pigeon	7	Other than crops (human activity area)	No	5	Yes (Maize)	Individual	5	Crop/gizzard & liver (1); liver (4)	39.7	1.1 ; 1.4 ; 0.6 ; <LoD (2)	Yes (acetylcholinesterase inhibitors; alphachloralose)	No	a : Proved (imidacloprid residues detected in digestive tract and liver)	b : Low (Cluster but not found in crop)	a : Strong (imidacloprid concentration in liver ≥ 1mg/kg)	Likely
1996	October	Yes	Grey partridge	2	Crops (sowing)	No	2	Yes (Cereals)	Individual	2	Crop/gizzard & liver	16.4 ; 17.0	0.6 ; 1.2	Yes (acetylcholinesterase inhibitors)	No	a : Proved (imidacloprid residues detected in digestive tract and liver)	a : Strong (Cluster found in crops)	a : Strong (imidacloprid concentration in liver ≥ 1mg/kg)	Very likely
1996	October	No	Grey partridge	1	Crops (sowing)	No	1	Yes (Cereals)	Individual	1	Crop/gizzard & liver	3.1	< LoD	Yes (acetylcholinesterase inhibitors)	No	a : Proved (imidacloprid residues detected in digestive tract)	b : Low (Single animal incident)	b : Low (Neither nervous disorders observed nor liver imidacloprid residues detected)	Possible
1996	October	Yes	Brown hare	2	Crops (sowing)	No	2	No	Individual	2	Liver	-	4.8 ; 7.2	No	-	a : Proved (imidacloprid residues detected in liver)	a : Strong (Cluster found in crops)	a : Strong (imidacloprid concentration in liver ≥ 1mg/kg)	Very likely
1996	October	No	Ring-necked pheasant	1	Not specified	No	1	No	Individual	1	Crop/gizzard & liver	1.0	0.3	Yes (acetylcholinesterase inhibitors; alphachloralose; organochlorins; pyrethroids)	No	a : Proved (imidacloprid residues detected in digestive tract and liver)	b : Low (Single animal incident)	b : Low (No nervous disorders observed & imidacloprid concentration in liver ≤ 1mg/kg)	Possible
1996	November	Yes	Pigeon sp.	2	Other than crops (human activity area)	Yes (paralysis)	2	Yes (Not specified)	Pool of individuals	-	Crop/gizzard	2.0	-	Yes (alphachloralose)	No	a : Proved (imidacloprid residues detected in digestive tract)	b : Low (Cluster but not found in crop)	a : Strong (nervous disorders observed)	Likely
1997	March	Yes	Wood-pigeon	5	Crops (sowing)	No	5	Yes (Cereals)	Individual	2	Crop/gizzard & liver (1); liver (1)	11.5	0.5 ; < LoD	Yes (acetylcholinesterase inhibitors)	Furathiocarb (Crop/gizzard; 18.0 ; 12.0)	a : Proved (imidacloprid residues detected in digestive tract and liver)	a : Strong (Cluster found in crops)	b : Low (other pesticides residue detected)	Likely
1998	October	Yes	Rock/Feral pigeon	30	Not specified	No	5	No	Individual	5	Crop/gizzard & liver	18.0 ; 28.0 ; 15.6 ; 19.0 ; 18.0	2.0 ; 0.6 ; 1.0 ; < LoD (2)	No	-	a : Proved (imidacloprid residues detected in digestive tract and liver)	b : Low (Cluster but not found in crop)	a : Strong (imidacloprid concentration in liver ≥ 1mg/kg)	Likely
1998	October	Yes	Rock/Feral pigeon	3	Other than crops (human activity area)	Yes (nervous disorders)	3	No	Individual	3	Crop/gizzard	15.3 ; 15.5 ; 9.2	-	No	-	a : Proved (imidacloprid residues detected in digestive tract)	b : Low (Cluster but not found in crop)	a : Strong (nervous disorders observed)	Likely

1998	October	Yes	Grey partridge	3	Crops (sowing)	No	3	No	Individual	3	Crop/gizzard & liver	33.0 ; 8.8 ; 22.5	< LoD (3)	No	-	a : Proved (imidacloprid residues detected in digestive tract)	a : Strong (Cluster found in crops)	b : Low (Neither nervous disorders observed nor liver imidacloprid residues detected)	Likely
1998	November	Yes	Grey partridge	5	Crops (plant growth stage not specified)	No	5	Yes (Cereals)	Individual	5	Crop/gizzard & liver	21.6 ; 17.8 ; 19.3 ; 1706.0 ; 17.5	< LoD (5)	No	-	a : Proved (imidacloprid residues detected in digestive tract)	a : Strong (Cluster found in crops)	b : Low (Neither nervous disorders observed nor liver imidacloprid residues detected)	Likely
1998	December	Yes	Rock/Feral pigeon	3	Other than crops (human activity area)	Yes (paralysis & red droppings)	3	No	Pool of individuals	-	Crop/gizzard	1.2	-	No	-	a : Proved (imidacloprid residues detected in digestive tract)	b : Low (Cluster but not found in crop)	a : Strong (nervous disorders observed)	Likely
1999	May	No	Partridge sp.	1	Crops (plant growth stage not specified)	No	1	Yes (Maize)	Individual	1	Not specified (positive)	-	-	No	-	b : Strongly suspected imidacloprid residues detected but matrix not specified	b : Low (Single animal incident)	b : Low (Neither nervous disorders observed nor liver imidacloprid residues detected)	Possible
1999	October	Yes	Grey partridge	2	Crops (plant growth stage not specified)	No	1	No	Individual	1	Crop/gizzard & liver	47.0	15.0	No	-	a : Proved (imidacloprid residues detected in digestive tract and liver)	a : Strong (Cluster found in crops)	a : Strong (imidacloprid concentration in liver ≥ 1mg/kg)	Very likely
1999	October	Yes	Partridge sp.	6	Crops (sowing)	No	6	Yes (Cereals)	Individual	6	Crop/gizzard (5); Crop/gizzard & liver (1)	2.6 ; 4.7 ; 3.4 ; 4.0 ; 4.0 ; <LoD	< LoD	Yes (fipronil)	Fipronil (Crop/gizzard; 87.0 ; 107.0 ; 88.0 ; 81.6 ; < LoD ; < LoD)	a : Proved (imidacloprid residues detected in digestive tract)	a : Strong (Cluster found in crops)	b : Low (other pesticides residue detected)	Likely
2000	February	Yes	Wood-pigeon	2	Other than crops (semi-natural area)	Yes (nervous disorders)	2	No	Individual	2	Crop/gizzard	1.2 ; 1.9	-	No	-	a : Proved (imidacloprid residues detected in digestive tract)	b : Low (Cluster but not found in crop)	a : Strong (nervous disorders observed)	Likely
2000	March	No	Grey partridge	1	Crops (plant growth stage not specified)	No	1	No	Individual	1	Crop/gizzard & liver	5.3	< LoD	Yes (acetylcholinesterase inhibitors)	No	a : Proved (imidacloprid residues detected in digestive tract)	b : Low (Single animal incident)	b : Low (Neither nervous disorders observed nor liver imidacloprid residues detected)	Possible
2000	March	No	Wood-pigeon	1	Other than crops (semi-natural area)	No	1	No	Individual	1	Crop/gizzard & liver	0.4	0.3	No	-	a : Proved (imidacloprid residues detected in digestive tract and liver)	b : Low (Single animal incident)	b : Low (No nervous disorders observed & imidacloprid concentration in liver ≤ 1mg/kg)	Possible
2000	March	No	Rock/Feral pigeon	1	Crops (plant growth stage not specified)	No	1	No	Individual	1	Crop/gizzard	2.2	-	Yes (acetylcholinesterase inhibitors)	No	a : Proved (imidacloprid residues detected in digestive tract)	b : Low (Single animal incident)	b : Low (Neither nervous disorders observed nor liver imidacloprid residues detected)	Possible
2000	April	No	Grey partridge	1	Crops (sowing)	No	1	Yes (Maize)	Individual	1	Crop/gizzard & liver	15.0	< LoD	No	-	a : Proved (imidacloprid residues detected in digestive tract)	b : Low (Single animal incident)	b : Low (Neither nervous disorders observed nor liver imidacloprid residues detected)	Possible
2000	April	No	Grey partridge	1	Crops (plant growth stage not specified)	No	1	No	Individual	1	Crop/gizzard & liver	< LoD	12.0	Yes (acetylcholinesterase inhibitors)	Acetylcholinesterase inhibitor (Crop/gizzard; 20.0)	a : Proved (imidacloprid residues detected in liver)	b : Low (Single animal incident)	b : Low (other pesticides residue detected)	Possible
2000	May	No	Wood-pigeon	1	Not specified	No	1	No	Individual	1	Crop/gizzard	5.1	-	Yes (acetylcholinesterase inhibitors)	No	a : Proved (imidacloprid residues detected in digestive tract)	b : Low (Single animal incident)	b : Low (Neither nervous disorders observed nor liver imidacloprid residues	Possible

																		detected)	
2001	October	No	Red-legged partridge	1	Crops (plant growth stage not specified)	No	1	No	Individual	1	Crop/gizzard	0.9	-	No	-	a : Proved (imidacloprid residues detected in digestive tract)	b : Low (Single animal incident)	b : Low (Neither nervous disorders observed nor liver imidacloprid residues detected)	Possible
2002	March	Yes	Rock/Feral pigeon	5	Other than crops (human activity area)	No	5	Yes (Cereals)	Pool of individuals	-	Crop/gizzard	7.1	-	Yes (acetylcholinesterase inhibitors)	Acetylcholinesterase inhibitor (Crop/gizzard; positive)	a : Proved (imidacloprid residues detected in digestive tract)	b : Low (Cluster but not found in crop)	b : Low (other pesticides residue detected)	Possible
2002	April	Yes	Rock/Feral pigeon	20	Crops (plant growth stage not specified)	No	5	No	Pool of individuals	-	Crop/gizzard	16.2	-	Yes (acetylcholinesterase inhibitors; fipronil)	Acetylcholinesterase inhibitor (Crop/gizzard; positive)	a : Proved (imidacloprid residues detected in digestive tract)	a : Strong (Cluster found in crops)	b : Low (other pesticides residue detected)	Likely
2002	October	Yes	Grey partridge	3	Crops (plant growth stage not specified)	Yes (nervous disorders)	3	Yes (Cereals)	Pool of individuals	-	Crop/gizzard	9.7	-	Yes (anticoagulants)	No	a : Proved (imidacloprid residues detected in digestive tract)	a : Strong (Cluster found in crops)	a : Strong (nervous disorders observed)	Very likely
2003	October	Yes	Grey partridge	2	Crops (sowing)	No	2	Yes (Cereals)	Pool of individuals	-	Crop/gizzard	7.8	-	Yes (fipronil)	Fipronil (Crop/gizzard; 7.3)	a : Proved (imidacloprid residues detected in digestive tract)	a : Strong (Cluster found in crops)	b : Low (other pesticides residue detected)	Likely
2003	October	Yes	Grey partridge	12	Crops (plant growth stage not specified)	No	12	Yes (Not specified)	Pool of individuals	-	Crop/gizzard	37.3	-	No	-	a : Proved (imidacloprid residues detected in digestive tract)	a : Strong (Cluster found in crops)	b : Low (Neither nervous disorders observed nor liver imidacloprid residues detected)	Likely
2003	November	Yes	Rock/Feral pigeon	60	Crops (sowing)	No	2	Yes (Cereals)	Pool of individuals	-	Crop/gizzard	11.7	-	No	-	a : Proved (imidacloprid residues detected in digestive tract)	a : Strong (Cluster found in crops)	b : Low (Neither nervous disorders observed nor liver imidacloprid residues detected)	Likely
2004	April	Yes	Pigeon sp.	2	Crops (sowing)	No	2	No	Individual	2	Crop/gizzard	8.8 ; 9.7	-	No	-	a : Proved (imidacloprid residues detected in digestive tract)	a : Strong (Cluster found in crops)	b : Low (Neither nervous disorders observed nor liver imidacloprid residues detected)	Likely
2004	April	Yes	Rock/Feral pigeon	10	Crops (sowing)	No	3	No	Individual	3	Crop/gizzard & liver	10.6 ; 12.0 ; 9.0	< LoD (2) ; 43.5	No	-	a : Proved (imidacloprid residues detected in digestive tract and liver)	a : Strong (Cluster found in crops)	a : Strong (imidacloprid concentration in liver ≥ 1mg/kg)	Very likely
2004	May	No	Rock/Feral pigeon	1	Other than crops (human activity area)	Yes (nervous disorders)	1	Yes (Maize)	Individual	1	Crop/gizzard & liver	8.3	< LoD	Yes (alphachloralose; fipronil)	No	a : Proved (imidacloprid residues detected in digestive tract)	b : Low (Single animal incident)	a : Strong (nervous disorders observed)	Likely
2004	May	No	Grey partridge	1	Crops (sowing)	Yes (nervous disorders)	1	Yes (Maize)	Individual	1	Crop/gizzard & liver	18.0	< LoD	No	-	a : Proved (imidacloprid residues detected in digestive tract)	b : Low (Single animal incident)	a : Strong (nervous disorders observed)	Likely
2004	May	Yes	Wood-pigeon	3	Other than crops (semi-natural area)	Yes (fall in flight)	1	No	Individual	1	Crop/gizzard	39.5	-	Yes (alphachloralose)	No	a : Proved (imidacloprid residues detected in digestive tract)	b : Low (Cluster but not found in crop)	a : Strong (nervous disorders observed)	Likely
2004	October	Yes	Grey partridge	3	Crops (sowing)	Yes (inability to fly)	3	Yes (Cereals)	Pool of individuals	-	Crop/gizzard & liver	8.8	< LoD	No	-	a : Proved (imidacloprid residues detected in	a : Strong (Cluster found in crops)	a : Strong (nervous disorders observed)	Very likely

																digestive tract)			
2004	November	No	Grey partridge	1	Not specified	Yes (nervous disorders)	1	Yes (Cereals)	Individual	1	Crop/gizzard & liver	6.0	< LoD	Yes (anticoagulants)	No	a : Proved (imidacloprid residues detected in digestive tract)	b : Low (Single animal incident)	a : Strong (nervous disorders observed)	Likely
2005	October	Yes	Rock/Feral pigeon	100	Not specified	Yes (nervous disorders)	1	Yes (Cereals)	Individual	1	Crop/gizzard	36.7	-	Yes (alphachloralose)	No	a : Proved (imidacloprid residues detected in digestive tract)	b : Low (Cluster but not found in crop)	a : Strong (nervous disorders observed)	Likely
2005	October	Yes	Grey partridge	4	Crops (plant growth stage not specified)	Yes (nervous disorders ; One bird released safe after ~12h)	3	No	Individual	3	Crop/gizzard & liver	6.9 ; < LoD (2)	7.6 ; 8.5 ; 4.8	No	-	a : Proved (imidacloprid residues detected in digestive tract and liver)	a : Strong (Cluster found in crops)	a : Strong (nervous disorders observed & imidacloprid concentration in liver ≥ 1mg/kg)	Very likely
2005	October	Yes	Grey partridge	2	Crops (plant growth stage not specified)	No	2	Yes (Cereals)	Pool of individuals	-	Crop/gizzard	11.5	-	Yes (acetylcholinesterase inhibitors; fipronil)	Acetylcholinesterase inhibitor (Crop/gizzard; positive)	a : Proved (imidacloprid residues detected in digestive tract)	a : Strong (Cluster found in crops)	b : Low (other pesticides residue detected)	Likely
2005	October	Yes	Rock/Feral pigeon	3	Other than crops (human activity area)	No	1	No	Individual	1	Crop/gizzard & liver	9.1	< LoD	No	-	a : Proved (imidacloprid residues detected in digestive tract)	b : Low (Cluster but not found in crop)	b : Low (Neither nervous disorders observed nor liver imidacloprid residues detected)	Possible
2005	November	Yes	Pigeon sp.	6	Other than crops (human activity area)	No	3	Yes (Cereals)	Pool of individuals	-	Crop/gizzard & liver	7.7	< LoD	Yes (alphachloralose)	Alphachloralose (Crop/gizzard; 64.5)	a : Proved (imidacloprid residues detected in digestive tract)	b : Low (Cluster but not found in crop)	b : Low (other pesticides residue detected)	Possible
2006	October	Yes	Rock/Feral pigeon	5	Other than crops (human activity area)	No	1	Yes (Cereals)	Individual	1	Crop/gizzard	16.2	-	Yes (alphachloralose)	No	a : Proved (imidacloprid residues detected in digestive tract)	b : Low (Cluster but not found in crop)	b : Low (Neither nervous disorders observed nor liver imidacloprid residues detected)	Possible
2007	October	Yes	Grey partridge	2	Crops (plant growth stage not specified)	Yes (nervous disorders)	2	Yes (Cereals)	Pool of individuals	-	Crop/gizzard	115.0	-	No	-	a : Proved (imidacloprid residues detected in digestive tract)	a : Strong (Cluster found in crops)	a : Strong (nervous disorders observed)	Very likely
2007	October	Yes	Stock dove	4	Other than crops (semi-natural area)	No	4	Yes (Cereals)	Pool of individuals	-	Crop/gizzard	63.0	-	No	-	a : Proved (imidacloprid residues detected in digestive tract)	b : Low (Cluster but not found in crop)	b : Low (Neither nervous disorders observed nor liver imidacloprid residues detected)	Possible
2007	October	Yes	Pigeon sp.	20	Other than crops (semi-natural area)	Yes (fall in flight)	3	Yes (Cereals)	Pool of individuals	-	Crop/gizzard	86.0	-	Yes (alphachloralose)	No	a : Proved (imidacloprid residues detected in digestive tract)	b : Low (Cluster but not found in crop)	a : Strong (nervous disorders observed)	Likely
2007	October	Yes	Partridge sp.	6	Crops (plant growth stage not specified)	No	6	Yes (Not specified)	Pool of individuals	-	Crop/gizzard	106.7	-	No	-	a : Proved (imidacloprid residues detected in digestive tract)	a : Strong (Cluster found in crops)	b : Low (Neither nervous disorders observed nor liver imidacloprid residues detected)	Likely
2007	October	No	Pigeon sp.	1	Other than crops (human activity area)	Yes (nervous disorders)	1	Yes (Cereals)	Individual	1	Crop/gizzard	37.0	-	No	-	a : Proved (imidacloprid residues detected in digestive tract)	b : Low (Single animal incident)	a : Strong (nervous disorders observed)	Likely
2007	October	Yes	Grey partridge	2	Crops (sowing)	No	2	Yes (Cereals)	Pool of individuals	-	Crop/gizzard	84.7	-	No	-	a : Proved (imidacloprid residues detected in	a : Strong (Cluster found in crops)	b : Low (Neither nervous disorders observed nor liver imidacloprid	Likely

																digestive tract)		residues detected)	
2008	October	Yes	Pigeon sp.	3	Crops (plant growth stage not specified)	No	2	No	Pool of individuals	-	Crop/gizzard	16.1	-	Yes (alphachloralose)	No	a : Proved (imidacloprid residues detected in digestive tract)	a : Strong (Cluster found in crops)	b : Low (Neither nervous disorders observed nor liver imidacloprid residues detected)	Likely
2010	March	No	Rock/Feral pigeon	1	Other than crops (human activity area)	No	1	Yes (Cereals)	Individual	1	Seeds (7.5)	-	-	Yes (anticoagulants)	No	b : Strongly suspected imidacloprid residues detected in seeds found close to the carcass	b : Low (Single animal incident)	b : Low (Neither nervous disorders observed nor liver imidacloprid residues detected)	Possible
2010	March	Yes	Pigeon sp.	7	Other than crops (semi-natural area)	No	7	Yes (Cereals)	Pool of individuals	-	Crop/gizzard	4.5	-	No	-	a : Proved (imidacloprid residues detected in digestive tract)	b : Low (Cluster but not found in crop)	b : Low (Neither nervous disorders observed nor liver imidacloprid residues detected)	Possible
2010	March	No	Grey partridge	1	Crops (sowing)	Yes (ataxia)	1	Yes (Beets)	Individual	1	Crop/gizzard	120.0	-	No	-	a : Proved (imidacloprid residues detected in digestive tract)	b : Low (Single animal incident)	a : Strong (nervous disorders observed)	Likely
2010	June	No	Wood-pigeon	1	Crops (plant growth stage not specified)	No	1	No	Individual	1	Crop/gizzard	2.4	-	Yes (pyrethroids)	No	a : Proved (imidacloprid residues detected in digestive tract)	b : Low (Single animal incident)	b : Low (Neither nervous disorders observed nor liver imidacloprid residues detected)	Possible
2010	October	Yes	Grey partridge	6	Crops (sowing)	Yes (ataxia; One bird released safe after few hours)	1	No	Individual	1	Crop/gizzard	2.5	-	Yes (acetylcholinesterase inhibitors; alphachloralose)	No	a : Proved (imidacloprid residues detected in digestive tract)	a : Strong (Cluster found in crops)	a : Strong (nervous disorders observed)	Very likely
2010	October	Yes	Wood-pigeon	14	Crops (sowing)	No	4	Yes (Cereals)	Pool of individuals	-	Crop/gizzard	105.0	-	No	-	a : Proved (imidacloprid residues detected in digestive tract)	a : Strong (Cluster found in crops)	b : Low (Neither nervous disorders observed nor liver imidacloprid residues detected)	Likely
2010	October	Yes	Rock/Feral pigeon	28	Crops (sowing)	No	5	Yes (Cereals)	Pool of individuals	-	Crop/gizzard	214.0	-	Yes (wide-range screening GC-MS)	No	a : Proved (imidacloprid residues detected in digestive tract)	a : Strong (Cluster found in crops)	b : Low (Neither nervous disorders observed nor liver imidacloprid residues detected)	Likely
2010	October	Yes	Eurasian collared dove	20	Crops (sowing)	No	3	No	Pool of individuals	-	Crop/gizzard	45.0	-	Yes (acetylcholinesterase inhibitors; anticoagulants)	No	a : Proved (imidacloprid residues detected in digestive tract)	a : Strong (Cluster found in crops)	b : Low (Neither nervous disorders observed nor liver imidacloprid residues detected)	Likely
2010	October	Yes	Grey partridge	3	Crops (sowing)	No	3	Yes (Cereals)	Individual	3	Crop/gizzard	105.0 ; 10.0 ; 11.0	-	Yes (acetylcholinesterase inhibitors)	No	a : Proved (imidacloprid residues detected in digestive tract)	a : Strong (Cluster found in crops)	b : Low (Neither nervous disorders observed nor liver imidacloprid residues detected)	Likely
2010	November	Yes	Wood-pigeon	5	Crops (sowing)	Yes (ataxia)	2	Yes (Cereals)	Individual	2	Crop/gizzard	34.7;45.0	-	No	-	a : Proved (imidacloprid residues detected in digestive tract)	a : Strong (Cluster found in crops)	a : Strong (nervous disorders observed)	Very likely

2011	February	Yes	Common crane	2	Crops (plant growth stage not specified)	Yes (apathy)	2	Yes (Cereals)	Pool of individuals	-	Crop/gizzard	4.3	-	Yes (acetylcholinesterase inhibitors)	Carbofuran (Crop/gizzard; 247)	a : Proved (imidacloprid residues detected in digestive tract)	a : Strong (Cluster found in crops)	b : Low (other pesticides residue detected)	Likely
2011	March	No	Grey partridge	1	Crops (plant growth stage not specified)	No	1	No	Individual	1	Crop/gizzard & liver	721.0	< LoD	Yes (pyrethroids)	No	a : Proved (imidacloprid residues detected in digestive tract)	b : Low (Single animal incident)	b : Low (Neither nervous disorders observed nor liver imidacloprid residues detected)	Possible
2011	October	Yes	Grey partridge	2	Crops (plant growth stage not specified)	Yes (nervous disorders)	2	Yes (Cereals)	Individual	2	Crop/gizzard & liver	1.2 ; 4.5	< LoD (2)	Yes (acetylcholinesterase inhibitors)	No	a : Proved (imidacloprid residues detected in digestive tract)	a : Strong (Cluster found in crops)	a : Strong (nervous disorders observed)	Very likely
2011	October	Yes	Grey partridge	3	Crops (sowing)	No	3	Yes (Cereals)	Pool of individuals	-	Crop/gizzard & liver	113.4	< LoD	No	-	a : Proved (imidacloprid residues detected in digestive tract)	a : Strong (Cluster found in crops)	b : Low (Neither nervous disorders observed nor liver imidacloprid residues detected)	Likely
2011	October	Yes	Pigeon sp. and Fringillidae (not specified)	3	Other than crops (human activity area)	No	3	Yes (Cereals)	Pool of individuals	-	Crop/gizzard & liver	52.8	1.2	No	-	a : Proved (imidacloprid residues detected in digestive tract and liver)	b : Low (Cluster but not found in crop)	a : Strong (imidacloprid concentration in liver ≥ 1mg/kg)	Likely
2011	November	No	Wood-pigeon	1	Crops (sowing)	No	1	Yes (Cereals)	Individual	1	Crop/gizzard & liver	85.3	< LoD	No	-	a : Proved (imidacloprid residues detected in digestive tract)	b : Low (Single animal incident)	b : Low (Neither nervous disorders observed nor liver imidacloprid residues detected)	Possible
2011	October	Yes	Rock/feral pigeon and grey partridge	20	Crops (sowing)	Yes (fall in flight)	3	Yes (Cereals)	Pool of individuals	-	Crop/gizzard & liver	48.0	0.8	No	-	a : Proved (imidacloprid residues detected in digestive tract and liver)	a : Strong (Cluster found in crops)	a : Strong (nervous disorders observed)	Very likely
2011	October	Yes	Red-legged and grey partridges	2	Crops (sowing)	No	2	Yes (Cereals)	Pool of individuals	-	Crop/gizzard	19.0	-	No	-	a : Proved (imidacloprid residues detected in digestive tract)	a : Strong (Cluster found in crops)	b : Low (Neither nervous disorders observed nor liver imidacloprid residues detected)	Likely
2011	November	Yes	Grey partridge	2	Crops (sowing)	Yes (nervous disorders	1	Yes (Not specified)	Individual	1	Crop/gizzard & liver	53.0	< LoD	No	-	a : Proved (imidacloprid residues detected in digestive tract)	a : Strong (Cluster found in crops)	a : Strong (nervous disorders observed)	Very likely
2011	October	Yes	Grey partridge	3	Crops (sowing)	Yes (nervous disorders)	2	Yes (Cereals)	Individual	2	Crop/gizzard	27.3 ; 16.5	-	No	-	a : Proved (imidacloprid residues detected in digestive tract)	a : Strong (Cluster found in crops)	a : Strong (nervous disorders observed)	Very likely
2012	October	No	Pigeon sp.	1	Crops (sowing)	No	1	Yes (Not specified)	Individual	1	Crop/gizzard	0.8	-	Yes (acetylcholinesterase inhibitors)	Methiocarb (Crop/gizzard; 3.7)	a : Proved (imidacloprid residues detected in digestive tract)	b : Low (Single animal incident)	b : Low (other pesticides residue detected)	Possible
2012	October	Yes	Pigeon sp.	5	Crops (sowing)	Yes (nervous disorders ; Two bird released safe after ~48h)	2	Yes (Not specified)	Individual	2	Crop/gizzard & liver	131.0 ; 126.0	< LoD (2)	Yes (acetylcholinesterase inhibitors)	No	a : Proved (imidacloprid residues detected in digestive tract)	a : Strong (Cluster found in crops)	a : Strong (nervous disorders observed)	Very likely
2012	November	Yes	Pigeon sp.	12	Crops (sowing)	Yes (apathy; Seven bird released safe after	2	Yes (Cereals)	Individual	2	Crop/gizzard & liver	90.0 ; 122.0	1.6 ; 1.8	No	-	a : Proved (imidacloprid residues detected in digestive tract and liver)	a : Strong (Cluster found in crops)	a : Strong (nervous disorders observed & imidacloprid concentration in	Very likely

						~48h)												liver ≥ 1mg/kg)	
2012	October	Yes	Wood-pigeon	10	Not specified	No	1	Yes (Cereals)	Individual	1	Crop/gizzard	Positive	-	Yes (alphachloralose)	Alphachloralose (Crop/gizzard; positive)	a : Proved (imidacloprid residues detected in digestive tract)	b : Low (Cluster but not found in crop)	b : Low (other pesticides residue detected)	Possible
2012	October	Yes	Pigeon sp.	12	Crops (sowing)	Yes (nervous disorders	1	No	Individual	1	Crop/gizzard	185.0	-	No	-	a : Proved (imidacloprid residues detected in digestive tract)	a : Strong (Cluster found in crops)	a : Strong (nervous disorders observed)	Very likely
2012	April	Yes	Pigeon sp. and black-headed Gull	4	Crops (plant growth stage not specified)	No	4	Yes (Cereals)	Pool of individuals	-	Crop/gizzard	1.4	-	Yes (anticoagulants)	No	a : Proved (imidacloprid residues detected in digestive tract)	a : Strong (Cluster found in crops)	b : Low (Neither nervous disorders observed nor liver imidacloprid residues detected)	Likely
2013	October	Yes	Pigeon sp.	2	Other than crops (human activity area)	Yes (nervous disorders)	2	Yes (Cereals)	Individual	2	Crop/gizzard	240.0 ; 61.0	-	No	-	a : Proved (imidacloprid residues detected in digestive tract)	b : Low (Cluster but not found in crop)	a : Strong (nervous disorders observed)	Likely
2013	September	No	Pigeon sp.	1	Not specified	No	1	Yes (Cereals)	Individual	1	Crop/gizzard	119.4	-	No	-	a : Proved (imidacloprid residues detected in digestive tract)	b : Low (Single animal incident)	b : Low (Neither nervous disorders observed nor liver imidacloprid residues detected)	Possible
2013	October	Yes	Pigeon sp.	3	Crops (plant growth stage not specified)	Yes (apathy)	3	Yes (Not specified)	Individual	3	Crop/gizzard	195.5 ; 72.2 ; 55.8	-	No	-	a : Proved (imidacloprid residues detected in digestive tract)	a : Strong (Cluster found in crops)	a : Strong (nervous disorders observed)	Very likely
2013	September	Yes	Rock/Feral pigeon	2	Other than crops (human activity area)	Yes (fall in flight)	2	Yes (Cereals)	Pool of individuals	-	Crop/gizzard	23.0	-	Yes (alphachloralose)	No	a : Proved (imidacloprid residues detected in digestive tract)	b : Low (Cluster but not found in crop)	a : Strong (nervous disorders observed)	Likely
2013	October	Yes	Grey partridge	4	Crops (sowing)	No	4	Yes (Cereals)	Pool of individuals	-	Crop/gizzard & liver	132.6	< LoD	No	-	a : Proved (imidacloprid residues detected in digestive tract)	a : Strong (Cluster found in crops)	b : Low (Neither nervous disorders observed nor liver imidacloprid residues detected)	Likely
2013	December	Yes	Pigeon sp.	5	Other than crops (human activity area)	Yes (nervous disorders)	5	Yes (Cereals)	Pool of individuals	-	Crop/gizzard & liver	34.0	11.7	No	-	a : Proved (imidacloprid residues detected in digestive tract and liver)	b : Low (Cluster but not found in crop)	a : Strong (nervous disorders observed & imidacloprid concentration in liver ≥ 1mg/kg)	Likely
2013	November	Yes	Pigeon sp.	5	Other than crops (human activity area)	Yes (fall in flight)	1	Yes (Not specified)	Individual	1	Not specified (0.46)	-	-	No	-	b : Strongly suspected imidacloprid residues detected but matrix not specified	b : Low (Cluster but not found in crop)	a : Strong (nervous disorders observed)	Possible
2013	October	Yes	Pigeon sp.	6	Not specified	No	2	Yes (Cereals)	Pool of individuals	-	Crop/gizzard	73.0	-	No	-	a : Proved (imidacloprid residues detected in digestive tract)	b : Low (Cluster but not found in crop)	b : Low (Neither nervous disorders observed nor liver imidacloprid residues detected)	Possible
2013	October	Yes	Pigeon sp.	100	Not specified	No	5	No	Individual	5	Crop/gizzard & liver	189.5 ; 179.0 ; 193.0 ; 206.5 ; 286.7	12.5 ; 1.9 ; 0.3 ; 3.1 ; 1.3	No	-	a : Proved (imidacloprid residues detected in digestive tract and liver)	b : Low (Cluster but not found in crop)	a : Strong (imidacloprid concentration in liver ≥ 1mg/kg)	Likely

2014	October	Yes	Partridge sp.	2	Not specified	No	2	Yes (Cereals)	Pool of individuals	-	Crop/gizzard & liver	71.8	6.2	No	-	a : Proved (imidacloprid residues detected in digestive tract and liver)	b : Low (Cluster but not found in crop)	a : Strong (imidacloprid concentration in liver ≥ 1mg/kg)	Likely
2014	October	Yes	Grey partridge	5	Crops (sowing)	Yes (fall in flight)	1	Yes (Cereals)	Individual	1	Crop/gizzard	58.5	-	Yes (anticoagulants; fipronil; pyrethroids)	No	a : Proved (imidacloprid residues detected in digestive tract)	a : Strong (Cluster found in crops)	a : Strong (nervous disorders observed)	Very likely
2014	October	Yes	Wood-pigeon	5	Crops (plant growth stage not specified)	Yes (inability to fly)	1	No	Individual	1	Crop/gizzard & liver	166.0	< LoD	No	-	a : Proved (imidacloprid residues detected in digestive tract)	a : Strong (Cluster found in crops)	a : Strong (nervous disorders observed)	Very likely
2014	September	Yes	Rock/Feral pigeon	7	Crops (sowing)	Yes (apathy)	3	No	Pool of individuals	-	Crop/gizzard	146.0	-	Yes (anticoagulants)	Bromadiolone (Crop/gizzard; 1.2)	a : Proved (imidacloprid residues detected in digestive tract)	a : Strong (Cluster found in crops)	b : Low (other pesticides residue detected)	Likely
2014	November	Yes	Rock/Feral pigeon and common starling	25	Other than crops (human activity area)	Yes (apathy)	3	No	Individual	2	Crop/gizzard	215.0 ; 184.0	-	No	-	a : Proved (imidacloprid residues detected in digestive tract)	b : Low (Cluster but not found in crop)	a : Strong (nervous disorders observed)	Likely
